# Exploring the future of land use and food security: A new set of global scenarios

**DOI:** 10.1371/journal.pone.0235597

**Published:** 2020-07-08

**Authors:** Olivier Mora, Chantal Le Mouël, Marie de Lattre-Gasquet, Catherine Donnars, Patrice Dumas, Olivier Réchauchère, Thierry Brunelle, Stéphane Manceron, Elodie Marajo-Petitzon, Clémence Moreau, Marc Barzman, Agneta Forslund, Pauline Marty

**Affiliations:** 1 DEPE, Institut national de recherche pour l’agriculture, l’alimentation et l’environnement (INRAE), Paris, France; 2 Agrocampus-Ouest, SMART-LERECO, Institut national de recherche pour l’agriculture, l’alimentation et l’environnement (INRAE), Rennes, France; 3 Centre de coopération internationale en recherche agronomique pour le développement (CIRAD), ART-DEV, Montpellier, France; 4 ART-DEV, Université de Montpellier, CIRAD, CNRS, Université de Montpellier 3, Montpellier, France; 5 Université de Perpignan Via Domitia, Montpellier, France; 6 Centre de coopération internationale en recherche agronomique pour le développement (CIRAD), CIRED, Montpellier, France; 7 Centre de coopération internationale en recherche agronomique pour le développement (CIRAD), CIRED, Nogent-sur-Marne, France; 8 Centre de coopération internationale en recherche agronomique pour le développement (CIRAD), Paris, France; 9 Département EcoSocio, Institut national de recherche pour l’agriculture, l’alimentation et l’environnement (INRAE), Rennes, France; CSIRO, AUSTRALIA

## Abstract

Facing a growing and more affluent world population, changing climate and finite natural resources, world food systems will have to change in the future. The aim of the Agrimonde-Terra foresight study was to build global scenarios linking land use and food security, with special attention paid to overlooked aspects such as nutrition and health, in order to help explore the possible future of the global food system. In this article, we seek to highlight how the resulting set of scenarios contributes to the debate on land use and food security and enlarges the range of possible futures for the global food system. We highlight four main contributions. Combining a scenario building method based on morphological analysis and quantitative simulations with a tractable and simple biomass balance model, the proposed approach improves transparency and coherence between scenario narratives and quantitative assessment. Agrimonde-Terra’s scenarios comprise a wide range of alternative diets, with contrasting underlying nutritional and health issues, which accompany contrasting urbanization and rural transformation processes, both dimensions that are lacking in other sets of global scenarios. Agrimonde-Terra’s scenarios share some similarities with existing sets of global scenarios, notably the SSPs, but are usually less optimistic regarding agricultural land expansion up to 2050. Results suggest that changing global diets toward healthier patterns could also help to limit the expansion in agricultural land area. Agrimonde-Terra’s scenarios enlarge the scope of possible futures by proposing two pathways that are uncommon in other sets of global scenarios. The first proposes to explore possible reconnection of the food industry and regional production within supranational regional blocs. The second means that we should consider that a ‘perfect storm’, induced by climate change and an ecological crisis combined with social and economic crises, is still possible. Both scenarios should be part of the debate as the current context of the COVID-19 pandemic shows.

## Introduction

Facing a growing and more affluent world population, changing climate and finite natural resources, world food systems will have to change in the future. A lively debate has emerged on which direction changes in these systems should take [1]. In the early 2010s, several studies promoted the sustainable intensification of agriculture for increasing food supplies to feed a growing population, without expanding the agricultural land area, which would be detrimental to climate and biodiversity [2–5]. Then, a series of work emphasized that changing consumption patterns and reducing food wastage in order to mitigate the rise in food demand and limit the negative impact of the global food system on natural resources and the environment should also be part of the solution. This wave of work, partly from the climate change research community, pointed out the major role of livestock production as a source of negative environmental effects and therefore promoted a significant reduction in animal-based food consumption [6–11].

Meanwhile, there was an on-going debate around the concept of sustainable intensification. Among other criticisms, it was questioned by the proponents of agroecology [12–13]. In this context, a set of studies focused on agroecological production systems, including organic agriculture, with a view to assessing whether this type of supply response would be an appropriate strategy for feeding the world sustainably [14–16]. The results suggest that, combined with a reduction in food wastage and reduced consumption of animal-based foods, agroecological production systems, including organic farming, could feed a growing population without increasing agricultural land use and therefore be part of a sustainable food future.

Alternatively, some authors started to claim that there is a close link between human health and environmental health through food diets, which affects both the incidence of overweight, obesity and non-communicable diseases (NCD) and natural resources and the environment. Work in this field advocates a global dietary transition towards healthier diets as an option to feed a growing population while keeping the global food system within a safe operating space for humanity [17–21]. These studies suggest that there are win-win scenarios involving healthier diets, reduced food loss and waste, and increased agricultural productivity, which would make it possible to reduce the adverse consequences of the global food system on health and mortality risk, and on the environment.

The above-mentioned studies do not provide sets of scenarios describing alternative futures of global agriculture and food systems. Most often, they consider one scenario (usually a business-as-usual type scenario) and alternative trends for one or several identified drivers (such as food diets, agricultural productivity, waste and loss, etc.). Furthermore, they usually assess quantitatively the impacts of the scenario under consideration and its alternatives but do not provide narratives explaining how agriculture and food systems would shift from current trends to alternative ones. Several foresight analyses have proposed such sets of scenarios, with more or less focus on the future of agriculture and food. These foresight analyses provide both narratives explaining the driving forces underlying the scenarios and assessments (quantitative and qualitative) of the consequences of the scenarios. The MEA (Millennium Ecosystem Assessment, [22]), SRES (Special Report on Emissions, [23]), SSP (Shared Socio-economic Pathways, [24]), Agrimonde [25] and FAO [26] scenarios are examples of such foresight analyses. The latter two sets of scenarios focus on the future of agriculture and food, while the first three have a larger scope (ecosystem changes in MEA and climate change in the SRES and SSP scenarios), but still with a key role for agricultural and food futures. All these foresight studies are also part of the debate on the sustainability of agriculture and food. But one may notice that none of them involves health aspects. In all the available scenario sets, diet changes are not directed towards nutritional and health aspects but are primarily aimed at reducing the environmental impacts of agriculture and food systems. As a result, assumptions about future dietary changes most often concentrate on the energy content of diets and on the share of animal-based foods.

Overall, this body of literature shows that the future of the global food system is highly complex and very uncertain. It also emphasizes the key role of land use in the sustainability of the global food system. Finally, it points out the need to jointly consider health and environmental issues. In this regard, exploring the future of food systems through the links between land use and food security (including nutritional aspects) appears a relevant way to connect both sustainable production and healthy consumption issues.

Building a new set of global scenarios linking land use and food security in order to help explore what could be the future of the global food system was precisely the aim of the Agrimonde-Terra foresight, led by both INRA and CIRAD from 2012 to 2016. This foresight study was intended to highlight the drivers that influence land-use patterns and their impacts on global food and nutrition security. In addition, and as noted above, there was a need to better incorporate nutritional and health issues in existing sets of scenarios of future global food systems. Consequently, Agrimonde-Terra proposed five contrasting scenarios of land use and food security in 2050 and this new set of global scenarios was the first incorporating a wide range of dietary changes with contrasting underlying nutritional and health issues. The Agrimonde-Terra method, scenarios and insights are described in detail in Le Mouël *et al*. [27].

Following up on the Agrimonde-Terra study, in this article we seek to highlight how the set of Agrimonde-Terra scenarios contributes to the debate on land use and food security and enlarges the range of possible futures of the global food system. To this end, we first report briefly on the Agrimonde-Terra method, scenarios and results, in order to provide the reader with sufficient and relevant background information. Then we explain where the set of global scenarios proposed by Agrimonde-Terra stands within the landscape of global scenario studies and what it adds.

We highlight the four main contributions of Agrimonde-Terra’s set of scenarios. Firstly, the method used to build and then quantify Agrimonde-Terra’s scenarios is an original coupled approach that links qualitative narratives and quantitative modelling, which is different from the approach most often used in global scenario studies. Secondly, Agrimonde-Terra’s scenarios comprise a wide range of alternative diets, including a healthy diet (similar to the one promoted in [21]), which accompany contrasting urbanization and rural transformation processes. This makes the Agrimonde-Terra scenarios quite original since they incorporate nutritional and health aspects and they emphasize the role of urban-rural relationships as regards the future of agriculture and food, both dimensions that are lacking in other sets of global scenarios. Thirdly, and as an alternative to other sets of global scenarios, Agrimonde-Terra proposes a third way between commonly used (market, economic or geopolitical) globalization and fragmentation pathways (e.g., MEA Global Orchestration and Order from Strength scenarios or SSP5 and SSP3 scenarios). In this scenario, supranational regional blocs shape food systems by promoting regional food culture and reconnect the food industry to regional production through the development of medium-size cities and small towns. Fourthly, contrary to other sets of global scenarios, Agrimonde-Terra involves a multi-crises scenario that explores the future of agriculture and food in a fragmented world undergoing an ecological crisis.

## Method

Foresight is not about predicting the future but is concerned with improving our understanding of future developments and the forces likely to shape them, and anticipating them with relevant actions [28–29]. The Agrimonde-Terra foresight study has a heuristic function whose challenge is to better understand the potential and risks of contemporary dynamics in agriculture and food systems, by exploring the possible futures of land use and food security. It also aims to contribute to strategic thinking, research and public debate.

**Fig 1:**
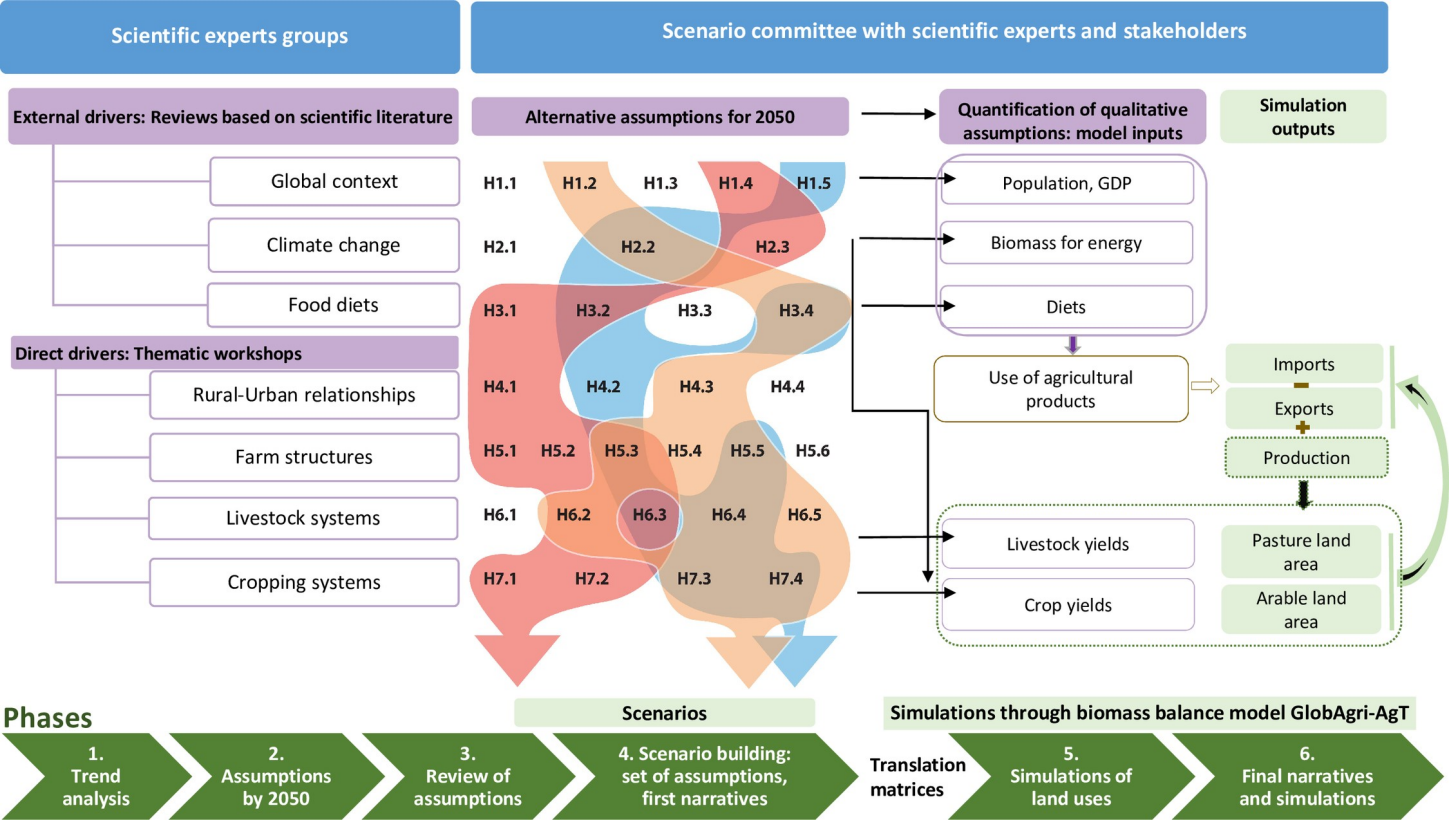
An overview of Agrimonde-Terra’s foresight method.

Agrimonde-Terra’s foresight method is a coupled approach combining scenario building and quantitative simulations (Fig 1). The scenario building process aims to ensure the consistency and plausibility of each scenario and to explore broad ranges of possible futures through contrasting scenarios, while quantitative simulations measure the scale and scope of changes described in the scenarios and provide elements for comparing them.

### Scenario design

Agrimonde-Terra’s foresight work used an exploratory methodology in order to deal with the uncertainties and complexities associated with global and inter-linked issues as well as non-linear changes in the land use and food security system. The scenario building approach of Agrimonde-Terra envisages what can or might happen, without reducing *a priori* the complexity of the system, neither in its structural dimensions nor in its temporal dynamics [30–31]. In contrast to most existing sets of global scenarios, Agrimonde-Terra’s scenarios were not developed along two axes that characterize the major and most uncertain driving forces or outcomes regarding the future of the system concerned. For example, the MEA scenarios were developed along two main driving forces: global governance for international cooperation and trade (globalized vs. regionalized), and attitudes towards ecosystem management (pro-active vs. reactive); the SSP scenarios were developed along two main outcomes: challenges for climate change mitigation, and challenges for climate change adaptation. According to some authors, the 2 × 2 scenario method can have “a restrictive scope and an overemphasis on some factors over others” [32–33]. Instead, we used a morphological analysis that provides a multidimensional systemic representation [29, 34].

Morphological analysis is “a method for structuring and investigating the total set of relationships contained in multi-dimensional, non-quantifiable problem complexes” [35–36]. Applied in the field of foresight studies, morphological analysis helps “to consider the entire field of possibilities and construct scenarios” [37–38]. First, the system under study and its main drivers are defined. Then, alternative assumptions of change are elaborated for each driver. The morphological table sets together these alternative assumptions per driver and thereby helps visualize and explore combinations of driver assumptions. The internal consistency of combinations is assessed, in order to “eliminate incompatible combinations… and create plausible combinations” [38]. The whole process is conducted with the implementation, at different phases of the study, of various forums (i.e. expert groups) to discuss the assumptions of change of drivers, the combinations of assumptions and their internal consistency, and the scenarios as retained plausible combinations of assumptions [39]. This systematic method makes it possible to investigate multiple plausible configurations, causal links and interactions between the different drivers of a system. Based on the knowledge of expert groups, it ensures the consistency and plausibility of scenarios [40].

As far as Agrimonde-Terra is concerned, the land use and food security system and its main drivers were first defined. Agrimonde-Terra considers that land-use changes result from complex interactions between direct and external drivers [41], and affect food security. The direct and external drivers considered are, respectively: cropping systems, livestock systems, farm structures, and urban-rural relationships (including urbanization); global (political, economic and social, including demography) context, climate change and food diets. Theses drivers are reported on the left-hand side panel of Fig 1.

Several expert groups were involved in the scenario building process (Fig 1). They provided knowledge and assessments about current trends and possible changes, and built collective intelligence about alternative futures. The whole study involved around 80 international experts, including scientific expert groups in the early stages (thematic workshops) and a scenario committee composed of 19 members (either scientists or stakeholders from international and national institutions as well as civil society) that provided guidance on scenario building (see Acknowledgements).

### The scenario development process

As shown in Fig 1, scenario building involved six phases (lower green blocks) with five different expert groups (four ‘scientific expert groups’ and one ‘scenario committee’, upper blue blocks).

**Fig 2:**
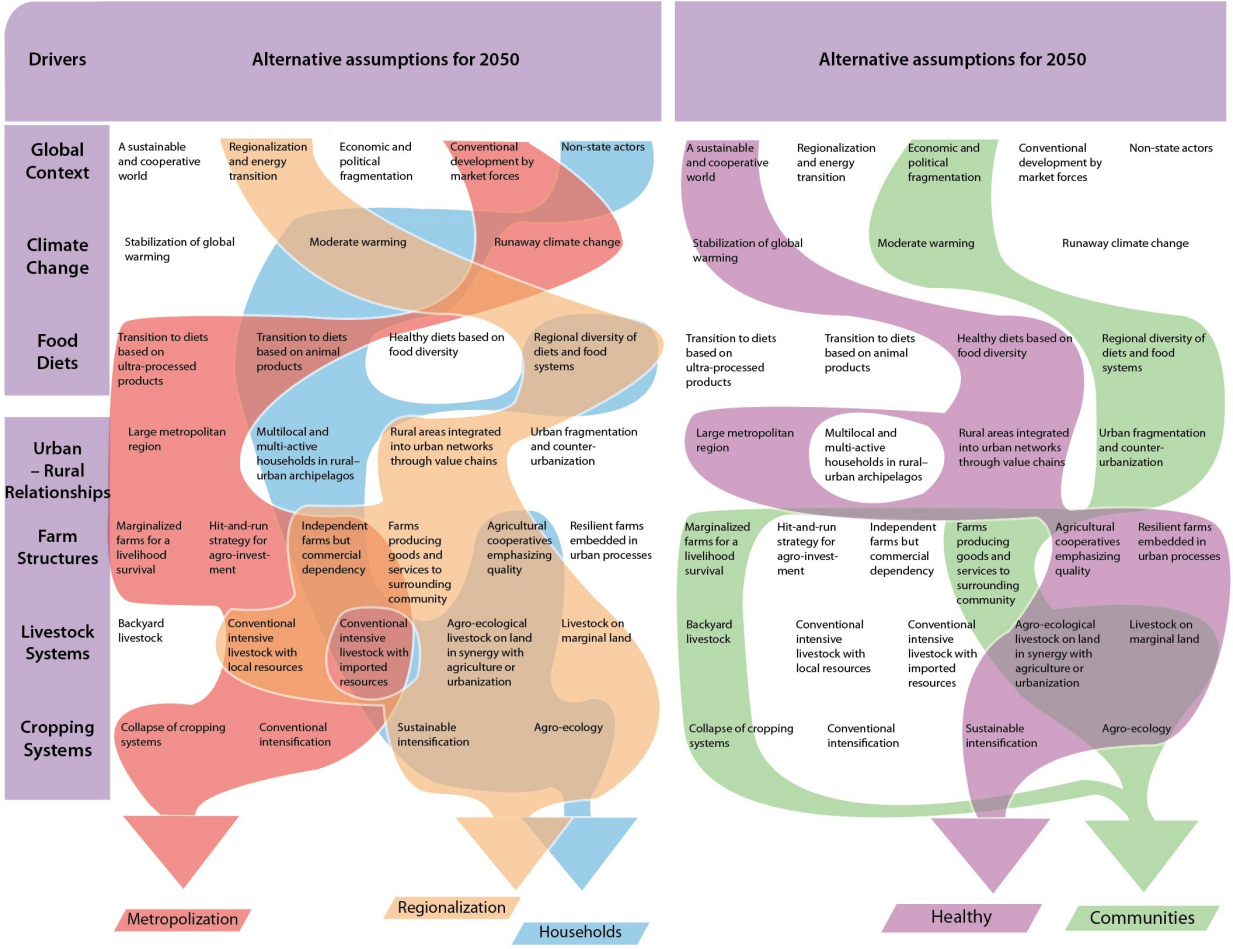
The five Agrimonde-Terra scenarios.

Phases 1 and 2 deal with the long-term dynamics of the drivers in the land use and food security system. They relate to the left-hand panel of Fig 1. The dynamics of direct and external drivers were analyzed through trend analyses with the objective of identifying past and current trends as well as potential disruptions that could shape their future development (Phase 1). In phase 2, alternative assumptions for the future of each driver were elaborated. They describe qualitatively different pathways to 2050 for each driver. Phases 1 and 2 were conducted through thematic workshops for the four direct drivers. Each workshop involved a specific group of academic researchers specialized in that driver. For each driver, two meetings were organized: the first aimed to discuss the past trends of evolutions, and the second dedicated to building alternative assumptions of future evolutions. For external drivers, the analysis was conducted by the project team. For all drivers, trend analyses and alternative qualitative assumptions for the future were based on experts’ knowledge, literature reviews and available data (including basic descriptive statistics on time series data for characterizing past and current trends). As a result of these two phases, alternative assumptions about possible changes by 2050 have been built for each driver; they are the ‘building blocks’ of the morphological table (see the graph in the central panel of Fig 1, and Fig 2; for each driver the alternative assumptions to 2050 are reported in the cells of the table).

The following two phases (3 and 4) deal with the elaboration of scenarios and narratives. They relate to the central panel of Fig 1. Five contrasting scenarios were built based on extensive discussions between academic researchers and stakeholders in the scenario committee. The scenario committee first assessed the alternative assumptions to 2050 for all drivers resulting from the previous phases. Then, it built contrasting scenarios mobilizing the morphological table. Each scenario combines one or several assumptions of change per driver (see the graph in the central panel of Fig 1, where scenarios are identified by the colored ribbons), respects causal relationships and seeks consistency across assumptions as well as plausibility. Each scenario describes the situation in land use and food security in 2050 and is developed into a narrative. Scenario narratives were drafted by the project team and discussed among experts of the scenario committee.

Phases 5 and 6 (right-hand panel of Fig 1) are concerned with the quantitative assessment of the scenarios.

### Scenario simulation

The quantitative impacts of scenarios in terms of land use and agricultural production and trade have been analyzed and discussed through an iterative process with the scenario committee.

Scenario simulations were conducted with GlobAgri-AgT [27]. The structure and functioning of the model are depicted in the right-hand panel of Fig 1 (and also in detail in the S1 File). For each agri-food product (including grass and various forage plants) in each world region, there is a resource-utilization equation. In plant product equations, the production component is linked to required land area (arable and pasture) through yield parameters, while the feed component is linked to animal production through feed-to-output parameters. In each equation, imports are fixed shares of domestic utilization while exports are fixed shares of the world market. The food and other uses components are exogenous in the model. Their levels, which are an input for the simulation model, result from assumed changes in demography, food diets and non-food use in the scenarios. Production and land use, feed and trade components are endogenous in the model. Their levels are calculated by the model given the changes in crop and livestock productivity assumed in the scenarios.

Each world region has a maximum cultivable area. When domestic needs change in one region, domestic production adjusts freely until the maximum cultivable area is reached. Then, additional needs are covered through trade: first, the region decreases proportionally all its export market shares; second, if not sufficient, the region increases proportionally all its import coefficients.

GlobAgri-AgT considers 38 agri-food products and 14 world regions (detailed in the S1 File). The reference year is the 2007–2009 average and the simulation horizon is 2050. Data used are mainly the FAO’s commodity balances [42]. Additional data are from Herrero *et al*. [43] for feed rations (including grass and forage), Monfreda *et al*. [44] for production and area of forage plants, and GAEZ [45] for maximum cultivable areas.

The starting point is the morphological table, which reports the alternative assumptions of change for each driver. These qualitative assumptions are first translated into quantitative model inputs. This involves establishing detailed translation matrices between global qualitative assumptions (for example, for the diet driver, ‘Transition to healthy diets based on food diversity’) and model input levels for each agri-food product and each world region (for example, food consumption per capita of wheat, fruit and vegetables, dairy or poultry meat in West Africa or India). The black arrows between the central and the right-hand panel of Fig 1 show these translation matrices. It is worth noting that these translation matrices do not rely on statistical methods using past observed dynamics of time series to make projections to 2050. They are based on experts’ opinions and existing literature (including available projections with various time horizons).

Once all qualitative assumptions for all the drivers have been translated into quantitative inputs for the model, scenarios may be simulated. As shown in the central panel of Fig 1, several assumptions of change patterns may co-exist for several drivers in our scenarios. When relevant, the same scenario was simulated using alternative assumptions of change for one or two drivers. Such scenarios variants concern either food diets or cropping and livestock systems (see Table 1). Finally, given that GlobAgri-AgT cannot deal with the main specificities of the ‘Households’ scenario (mobility, multi-activity and networking), no quantitative results were provided for this scenario.

**Table 1:** Quantitative model inputs for scenarios’ simulation.

Scenarios *Drivers*	Initial	Metropolization		Regionalization		Healthy		Communities	
Model input variables	2007–09	2050		2050		2050		2050	
***Global context***									
Population (billion)	6.7	9.7							
***Climate change (CC)***		*Runaway*		*Moderate*		*Stabilization*		*Moderate*	
Impact on crop yields (%)		CC impacts on crop yields in 2050							
Wheat	-	-13		-6		0		-6	
Maize	-	-8		-4		0		-4	
Rice	-	-13		-7		0		-7	
Soyabean	-	-20		-10		0		--10	
Sugar plants	-	+13		+7		0		+7	
Pulses	-	-15		-7		0		-7	
Fruits&vegetable	-	-11		-6		0		-6	
Impact on cultivable area	(GAEZ 1–4^1^, million ha)								
	4341	4459		4400		4341		4400	
***Food diet***		*Transition*		*Regional*		*Healthy*		*Regional*^2^	
Daily calories/cap (kcal)	2802	3132		2785		2843		2507	
Diet pattern (% share)		Ultrap^3^	Animp^3^						
Meat	7.8	6.3	11.0	6.9		6.1		6.9	
Dairy&eggs	6.0	6.3	7.8	6.3		5.7		6.3	
Aquatic animals	1.2	1.2	1.1	1.3		1.1		1.3	
Pulses	2.6	1.3	2.8	3.6		7.1		3.6	
Wheat, maize, rice	42.7	40.8	37.0	40.4		34.5		40.4	
Other cereals	3.0	0.8	4.0	5.3		12.6		5.3	
Fruits&vegetable	6.3	5.1	6.7	6.2		15.0		6.2	
Roots&tuber	6.1	6.0	6.4	8.9		3.7		8.9	
Sugar&sweeteners	8.3	13.0	7.7	7.1		2.5		7.1	
Vegetable oils	10.0	14.0	10.0	5.8		8.3		5.8	
Other products	6.0	5.2	5.5	8.2		3.4		8.2	
***Cropping systems***		*Conventional intensification*	*A*^4^	*B*^4^	*C*^4^	*D*^4^	*AE*^4^	*Collapse*^4^
Crop yields (ton/ha)	Crop yields not including climate change impacts								
Wheat	2.8	4.3		3.6	3.4	3.4	3.3	3.1	2.8
Maize	5.3	8.7		7.1	6.6	7.1	6.6	6.0	5.3
Rice	4.2	4.6		4.4	4.4	4.4	4.3	4.3	4.2
Soyabean	2.5	3.2		3.2	3.0	2.8	2.7	2.7	2.5
Sugar plants	68.5	90.2		78.1	75.7	76.2	74.3	72.8	68.5
Pulses	0.9	1.3		2.0	1.7	2.5	2.1	1.2	0.9
Fruits&vegetable	14.3	19.7		21.5	19.7	30.1	26.2	17.6	14.3
***Livestock systems***		*Conventional intensification*		*A*^4^	*B*^4^	*C*^4^	*D*^4^	*AE*^4^	*Collapse*^4^
Feed to output ratios	Feed to output ratios in kg dry matter feed per kg output of animal product								
Bovine meat	66.8	42.9		42.9	43.0	43.0	43.0	43.0	60.6
Small ruminant meat	38.0	23.6		23.6	23.2	23.2	23.2	23.2	36.1
Dairy	3.3	2.9		2.9	2.9	2.9	2.9	2.9	3.2
Pork meat	5.4	5.0		5.0	5.4	5.4	5.4	5.4	5.4
Poultry meat	4.4	3.9		3.9	4.4	4.4	4.4	4.4	4.4

^1^GAEZ 1–4: GAEZ land categories 1 to 4, based on the Suitability Index (see S1 File).

^2^The ‘Communities’ scenario uses the regional food diet assumption but in a context of lower incomes per capita.

^3^The ‘Metropolization’ scenario was simulated with two alternative food diet assumptions: Transition towards diets based on ultra-processed products (Ultrap variant) and Transition towards diets based on animal products (Animp variant).

^4^The ‘Regionalization’, ‘Healthy’ and ‘Communities’ scenarios were simulated with two alternative cropping and livestock systems assumptions: Sustainable intensification for cropping systems and conventional intensification for livestock systems (technology variant A); Agroecology for cropping and livestock systems (technology variants B and D); Sustainable intensification for cropping systems and agroecology for livestock systems (technology variant C); Agroecology for cropping and livestock systems, but in a context of lower R&D investments (AE variant); Collapse of cropping systems and backyard livestock (Collapse variant).

### Agrimonde-Terra scenarios

Agrimonde-Terra offers a set of five scenarios, which are depicted in Fig 2, and whose narratives are summarized below. The corresponding detailed narratives are available in Mora [46]. Three scenarios, ‘Metropolization’, ‘Regionalization’ and ‘Households’, are based on current competing trends identified in most regions of the world. Two scenarios, ‘Healthy’ and ‘Communities’ involve potential breaks that could change the entire land use and food security system.

#### Land use driven by metropolization [‘Metropolization’ scenario]

By 2050, two-thirds of the world’s population lives in cities and more than 15% of the urban population lives in megacities (more than 10 million inhabitants). The world economy follows a conventional development based on fossil fuels. It is built upon a global network of megacities, which attract rural migrants, and concentrate populations and activities in industry, services and knowledge. As environmental concerns have taken a back seat, climate change has significant effects, especially in agriculture.

Processing, retailing and wholesaling transnational corporations control the greater part of food markets in both urban and rural areas. Two dynamics in diet change are occurring: one driven by the expansion of globalized value chains providing low-price ultra-processed foods (Ultrap variant), based on edible plant oils, refined cereals, sugars, salt and animal processed meat; the other variant supported by the major consumption of animal products, meat in particular (Animp variant), based on the increasing demand of affluent populations.

Agriculture is based on conventional intensification with high levels of inputs.

As a result of the evolution in the food system, unhealthy diets have led to a dramatic increase in diet-related non-communicable diseases in developed and developing countries, with growing food inequalities within urban areas and between urban and rural areas. Despite the increase in global trade and because of strong pressure on agricultural land as well as a regional specialization of agriculture at the global scale, considerable impacts of climate change on agriculture have made the food supply system more vulnerable, triggering occasional food crises, especially for low-income households.

#### Land use for regional food systems [‘Regionalization’ scenario]

By 2050, political and economic governance in supranational regional blocs arose as a way to address a series of issues such as financial crises, unemployment, pollution and high rates of non-communicable diet-related diseases. Within these blocs, States are managing energy transition and improving food diversity. They seek greater energy autonomy by increasing the production of renewable energy and by using regionally available fossil fuel resources. Regions applied the concept of ‘food sovereignty and subsidiarity’, wherein as much food as possible is produced within the region and the remaining share is imported. Medium-size cities and small towns became part of regional development, playing a significant role as intermediates between rural areas and larger cities.

Regional blocs shaped food systems by promoting regional food culture, investing preferentially in and reconnecting the food industry to regional production. Medium-size cities and small towns developed industrial and small-scale food processing. This had a positive knock-on effect on employment and income in agriculture and rural areas.

In a context of moderate climate change, diverse crop and livestock systems co-exist, from conventional systems to sustainable intensification or agroecology. Diversification and the search for more autonomy led to making cropping systems more agroecological, with varieties best suited to regional agri-climatic conditions, while also strengthening ties between crop and livestock systems. Depending on the region, cropping systems evolved towards sustainable intensification or agroecology, while livestock systems adopted conventional intensification, based on domestically produced animal feed, or agroecology pathways (technology variants A or B).

With the development of regional food value chains, nutrition transition towards the consumption of ultra-processed foods was limited, and food access for rural populations was improved. Globally, the regionalization of diets and food systems contributed to limiting international trade which, nevertheless, remains a major concern for net importing regions such as the Near and Middle East, North Africa and West Africa.

#### Land use for multi-active and mobile households [‘Households’ scenario]

In a highly globalized world, where people are highly mobile and public and private interests hybridized, non-State actors including civil society groups, international NGOs, local authorities, multinational companies, academic institutions, foundations and cities shaped social, economic and geopolitical transformation processes. They have organized themselves to form ad hoc networks that play a key role in trade and are gradually superseding sovereign governments. In this dynamic but unstable economic context, many households have improved and diversified their incomes by being much more mobile. Reversible, temporary, short- or long-distance mobility between rural and urban areas has driven social networks and economic strategies.

Diverse demands have drawn public attention to farming practices and farmers’ groups regarding health, biodiversity, the environment and climate change. Farming households are driving organizational and technical innovations in food value chains, networking with each other, via digital platforms that disintermediate and shorten traditional supply chains. Access to these platforms and their modes of regulation have become central to food governance.

Households increased and diversified their incomes by being located in both rural and urban areas and carrying out farming and non-farming activities. As networks diversified, ranging from local, regional and national scales to international, they became the basis for the development of agriculture. The intensification of cropping and livestock systems varies from highly technical systems with low environmental impacts that make great use of cutting-edge technologies (information technologies) to innovative techniques such as agroecological approaches in response to public demand.

Multi-activity contributes to ensuring food and nutrition security for rural and urban households by diversifying their income and guaranteeing direct access to foodstuffs.

#### Land use for food quality and healthy nutrition [‘Healthy’ scenario]

In the 2020s, as healthcare systems were saddled with the considerable costs associated with diet-related non-communicable diseases and, more generally, as the consequences of malnutrition on public heath were increasingly felt, most States implemented a raft of policy measures to shift consumption patterns towards healthier diets. These policies were aligned with international measures to deal with climate change by focusing on energy, transport, construction, food systems and carbon storage. Synergies across multiple scales (national, regional and international) were sought for food, agriculture and climate policies. Global soil improvement policies have also led to the rehabilitation of degraded land for agricultural use and carbon storage. National States and urban authorities shaped more inclusive development processes linking rural to metropolitan areas, improving transport and communication infrastructures, land planning and reducing losses and waste in food supply chains.

By 2050, to meet nutrition targets, food chains reshaped to promote access to diverse and healthy products such as fruits and vegetables, coarse grains and pulses, and to improve the nutritional quality of processing by preserving micronutrients and fibres. Access to fresh produce improved with the development of a large range of distribution channels such as outdoor markets, small retailers and large supermarkets.

To meet the challenges posed by under- and overnutrition, both crops and cropping systems have diversified, incorporating techniques from agroecology. Livestock systems are now re-associated with crop production in order to improve mineral cycles. Depending on the availability of capital and the situation in the agricultural labour force, cropping systems have evolved towards sustainable intensification (technology variant C) or agroecology (technology variant D).

As a result of the mixed cross-sectoral policies reshaping food markets and agriculture, global diets are much healthier than 40 years ago. The increase in unhealthy food consumption has been halted, and undernutrition decreased due to food diversification and better resilience of farm systems. International trade, which is now based on nutrition standards, is still significant.

#### Land as commons for rural communities in a fragmented world [‘Communities’ scenario]

By 2050, simultaneous financial, energy, ecological and geopolitical crises have shaped a world situation that is fragmented not only politically, but also economically. Unemployment increased thereby impeding metropolitan growth, generating an urban de-concentration. Reduced migration to metropolitan areas led to the growth of smaller towns and fragmented urban development, and to an increase of rural populations in some world regions (South Asia and sub-Saharan Africa). By 2050, food supply chains in urban areas rely on formal and informal markets for staple foods and on networks between urban communities and rural ones. Urban and peri-urban agriculture provides incomes and food for poorer urban households.

Due to this situation, changes in land use are highly diversified between one region and another depending on the different challenges they face (energy, climate, soils and water) and the collective ability or inability of farmers to bring about a transition to agroecology. Two evolutions have prevailed. In order to cope with multiple crises, in some places farmers have succeeded in organizing themselves within their community to develop agroecological farms (Agroecology variant). Delivering foodstuffs, energy and environmental services, agroecological agriculture is regarded as a central element of the food system and of social organization, ensuring a certain level of food and nutrition security for rural and urban communities. In other places, where farmers failed to organize themselves, subsistence farming based on conventional intensification generated collapse and stagnation in cropping yields (Collapse variant). Cropping systems encountered two types of pitfall, depending on the region, the demographic trends of rural populations and access to inputs: over-exploitation of the soil and over-intensification of small-scale farming generating strong adverse impacts on the environment. In this situation, due to the vulnerability of conventional intensification systems, regions with subsistence farming face repeated instances of food insecurity and contribute to deforestation.

### Agrimonde-Terra’s results regarding land use and food security

The land-use change impacts of scenarios, obtained from GlobAgri-AgT, provide information on the sustainability of the food systems involved in each scenario. They are also indicators of the ability of each scenario to ensure world food availability: the expansion in agricultural land area suggests increased tensions over land, which in turn puts food availability into question at both the world and regional level. The other three dimensions of food security (access, utilization and stability) and nutritional aspects are dealt with through a qualitative analysis, based on information provided by the scenario narratives (see also S1 Table). We present the results only at the world level. Detailed results for world regions or for specific products are available in Le Mouël *et al*. [27] (see also S2 File and S1 Fig).

### From morphological table to quantitative model inputs

The morphological table reports the alternative change assumptions for each driver through 2050. These are qualitative assumptions. For each driver, we developed translation matrices providing general rules for translating each qualitative assumption to 2050 into quantitative model inputs. These translation matrices are provided in S2 File. In order to illustrate our approach, and as background information for a better understanding of simulation results, we provide in Table 1 the resulting quantitative model inputs for each driver under alternative change assumptions to 2050, in world average (model inputs are different from one region to another) and for selected agri-food products. Scenarios involve contrasting sets of model’s inputs and the resulting range of scenarios’ simulations shows how the model responds to widely different inputs.

Some of the global scenario sets available assume that population change is sensitive to the global geopolitical, economic and social context, and differentiate population assumptions according to the scenario (for example, [47] for the SSPs). However, Agrimonde-Terra decided to retain a unique assumption of population change up to 2050 [48]. We acknowledge that this is a restrictive assumption. In counterpart, our assumption of constant population change across scenarios ensures that simulated impacts are due only to assumed changes in food diets and agricultural production systems (i.e., changes in food systems per se), and not to changes in population growth.

The climate change assumptions used in Agrimonde-Terra’s scenarios are differentiated through their impacts on crop yields and cultivable area in 2050. The Runaway climate change assumption (Runaway in Table 1) corresponds to RCP 8.5 (Representative Concentration Pathway, IPCC fifth assessment report) and has strong impacts on crop yields and cultivable area. In contrast, the Stabilization in climate warming assumption (Stabilization) corresponds to RCP 2.6 and has no impact. The Moderate warming assumption (Moderate) implies medium impacts on crop yields and cultivable area compared to the other climate change assumptions.

The food diets used in Agrimonde-Terra’s various scenarios differ in both their energy content and dietary pattern. Alternative diets in 2050 range from diets with high energy content and low to medium diversity (Ultrap: transition based on ultra-processed foods; Animp: transition based on animal products) to diets with a medium energy content and medium to high diversity (Regional: transition based on regional food heritage; Healthy: transition based on diverse and fresh foods) (see also the Discussion).

The alternative assumptions for changes in cropping systems imply differentiated levels of crop yields in 2050 that result from different levels of yield gap reduction, combined with a positive response of yields to trends in world demand (i.e., induced technical change, see S2 File). Cropping system assumptions also involve differentiated levels of cropping intensity. On average, the Conventional intensification assumption induces higher crop yields in 2050 than the Sustainable intensification assumption, followed by the Agroecology assumption. At this stage it is important to underline that crop yields in 2050, used as quantitative model inputs for each scenario, result from both the cropping system and climate change assumptions used in each scenario.

Feed-to-ouput ratios in the various livestock sectors measure the performance of production systems in transforming plant products into animal products. As a world average, the Conventional intensification assumption leads to decreasing feed-to-output ratios, suggesting improved performance in livestock systems. The improvement in performance is lower under the Agroecological livestock assumption and even lower under the Backyard livestock assumption.

### Model outputs: Contrasting land use futures

**Fig 3:**
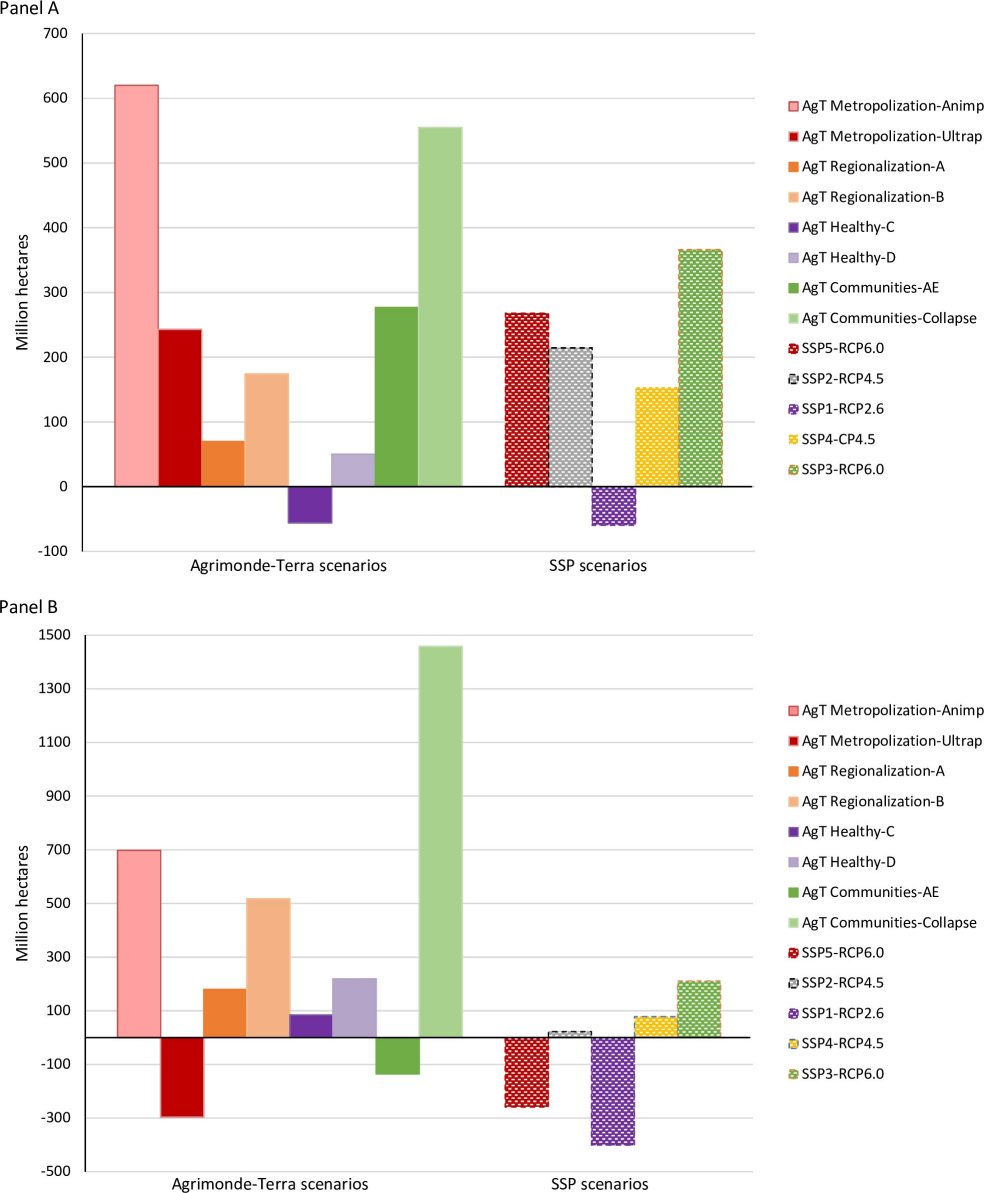
Consequences of the Agrimonde-Terra and SSP scenarios on world cropland area (Panel A) and world pastureland area (Panel B). Sources: GlobAgri-AgT simulation results and SSP database [49–50] (https://tntcat.iiasa.ac.at/SspDb/dsd?Action=htmlpage&page=20). Agrimonde-Terra scenarios include the impacts of differentiated pathways of climate change (Table 1). They are thus compared to SSP/RCP (Radiative Concentration Pathway) combinations of similar mitigation levels (respectively, RCP2.6, RCP4.5 and RCP6.0). The RCP8.5 option would fit better for comparison of SSP5 with Metropolization, however results for SSP5-RCP8.5 do not exist in the SSP database; Regarding SSP-RCP results, those reported are REMIND-MAGPIE-SSP5-RCP6.0, MESSAGE-GLOBIOM-SSP2-RCP4.5, IMAGE-SSP1-RCP2.6, GCAM4-SSP4-RCP4.5, AIM/CGE-SSP3-RCP6.0.

Most Agrimonde-Terra scenarios lead to an expansion of the world’s agricultural land area (Fig 3, agricultural land area is the sum of cropland and pastureland areas).

However, the extent of the expansion in world agricultural land varies widely across scenarios. It is particularly high for scenarios involving either a stagnation in crop yields and livestock production performance (+2 billion ha or +41% for ‘Communities with collapse’, Communities_Collapse) or a huge increase in animal product consumption (+1.3 billion ha or +27% for ‘Metropolization with animal products’, Metropolization_Animp). It is far more limited, even close to zero, for scenarios involving either reduced calorie availability in food regimes (+142 million ha or +3% for ‘Communities with agroecology’, Communities_AE) or a limited increase in the consumption of animal products, combined with a switch from ruminant to monogastric meat in meat consumption (+29 million ha or +0.6% for ‘Healthy with agricultural technology C’, Healthy-C, and -54 million ha or -1% for ‘Metropolization with ultra-processed products’, Metropolization_Ultrap).

Only the Metropolization_Ultrap and Healthy_C scenarios are able to produce sufficient food for the expected growing population up to 2050 without a significant expansion in the world agricultural land area and potential further major deforestation at the world scale. Under all the other scenarios, ensuring food availability in 2050 would likely result in large areas of deforestation all over the world. In some cases, the expansion in agricultural area is so huge that the scenarios concerned may be considered to be clearly unsustainable in 2050: Communities_Collapse and Metropolization_Animp scenarios, for example. However, one could also question the feasibility of the Regionalization scenario, which induces a significant expansion in the agricultural area: +249 million ha and up to +691 million ha in the A and B variants respectively. Although more limited, the expansion in the world agricultural area induced under the Healthy_D scenario (+269 million ha) puts into question the coherence of this scenario given that it involves strong mitigation objectives designed to stabilize climate change. There is clearly a consistency issue in the Healthy_D scenario between the deforestation it potentially induces and the mitigation objectives it involves, at the world level [51].

As reported in the introduction, overall our results are in line with the on-going debate on the future of world agriculture and food. The ‘Metropolization’ scenario and both of its food diet variants show that reducing animal-based food consumption is a powerful lever for feeding an increasing world population without expanding world agricultural land. The technology variants of the ‘Regionalization’ and ‘Healthy’ scenarios clearly illustrate the issue of sustainable intensification versus agroecology. Of course, our results in terms of the expansion in world agricultural land under both variants of both scenarios reflect our quantitative assumptions on the evolution in crop and animal yields to 2050 under both types of production systems. Agrimonde-Terra adopted rather conservative yield assumptions (cf. Discussion), which largely explain the fact that our results show a difficulty in feeding the growing population in 2050 without expanding world agricultural land if world agriculture changes towards agroecology. Indeed, we note that our results do not contradict those obtained by Muller *et al*. [16] but are clearly less optimistic. Finally, the ‘Healthy’ scenario suggests that moving towards healthy diets could be a win-win option for health and for limiting the expansion in agricultural land at the world level.

### Insights for food security and nutrition

The consequences of Agrimonde-Terra’s scenarios for world food security, in its four dimensions according to the FAO definition, are detailed in S1 Table, as well as their impacts in terms of nutrition.

Two scenarios are clearly not able to ensure world food and nutrition security in 2050: the ‘Metropolization’ and the ‘Communities’ scenarios, notably under the Collapse variant. Furthermore, two have ambiguous results: the ‘Regionalization’ and ‘Households’ scenarios. Only the ‘Healthy’ scenario seems likely to be able to meet the objective of world food and nutrition security in 2050.

‘Healthy’ is the scenario which contributes most to reducing not only overnutrition and related diseases, but also undernutrition, through diversified diets based on increased consumption of fruits and vegetables, coarse grains and pulses. This scenario is also the one which makes it possible to achieve world food availability at the cost of a rather limited expansion in the global agricultural land area. However, there are some regions where, according to our assumptions, promoting healthier diets induces an increased consumption of animal products, such as in India, West Africa and East Central and South (ECS) Africa. In these regions, even the ‘Healthy’ scenario is likely to induce an expansion in the agricultural land area and significant deforestation (S1 Fig). As the ‘Healthy’ scenario also involves a strong commitment to mitigating climate change, requiring the production of renewable energy and the maintenance of world forest cover as far as possible, there are potential tensions between the objectives of food security and climate change stabilization, resulting in increased competition for land between agricultural and forest use.

Conversely, ‘Metropolization’ is the scenario that contributes most to the expansion of overweight, obesity and diet-related non-communicable diseases all over the world, with considerable impacts on public health. In setting up a kind of race between changing food systems, increasing yields, deteriorating natural resources and propagating diet-related non-communicable diseases, ‘Metropolization’ induces a series of effects, which work against food and nutrition security at various levels (see S1 Table for more details).

‘Communities’ is a multi-crises scenario in which the deterioration in the performance of agricultural production would create a reduction in food availability at both world and regional levels. Because every region needs more land to meet its food needs, there is a struggle for resources, with very serious tensions over land and a degradation of natural resources, including soils. Long-term world food availability is thrown into question. In developed countries, this reduction in food availability could contribute to reducing overnutrition and related diseases. In developing countries, however, it could cause increased undernutrition (see S1 Table for more details).

The ‘Regionalization’ scenario induces a series of changes towards enhancing world and regional food and nutrition security, but at the same time leads to ambiguous results with regards to world food availability. In many cases, creating food systems aligned with traditional diets and based on diverse plant crops could actually improve the nutritional quality of local diets. This scenario also involves the development of agri-food industries in small and medium-sized cities. These industries positively affect rural development, rural employment and rural incomes. So, through the development of regional food value chains, ‘Regionalization’ may improve access to food for rural populations. However, ‘Regionalization’ is only able to ensure world and regional food availability at the cost of a significant expansion in the agricultural land area and potential considerable deforestation at the world level and for some regions (S1 Fig).

Finally, ‘Households’ appears to be an intermediate scenario, contributing to a decrease in undernutrition but with the opposite effect on overnutrition. ‘Households’ is also an intermediate scenario with regards to food and nutrition security, sharing common elements with three other scenarios. With ‘Healthy’ it shares a major public interest in nutrition, but without any State regulation. With ‘Regionalization’ it shares the idea that ‘more local’ food systems could respond to demands from consumer groups; the food diversification involved could have a positive impact on overnutrition. However, the main question raised by such a scenario is the extent to which changes in demand towards healthier foods from various consumer groups are able to induce transformations in food systems without a regulatory framework. It therefore seems plausible that the consumption of ultra-processed foods increases in this scenario, contributing to growth in overnutrition.

## Discussion

In this section, we discuss what the Agrimonde-Terra method and scenarios add to existing global scenario studies and to the current debate on land use and food security.

### Morphological analysis as a tool for linking qualitative narratives and quantitative impacts of scenarios

Agrimonde-Terra’s foresight method is an original approach linking qualitative scenarios and quantitative projections. In a recent review lead by Wiebe *et al*. [52], coupled approaches are defined as a “current research front” and “a new gold standard beyond integrated modelling approaches such as SAS” (Story And Simulation approach), in order to deal with “increasingly complex environmental and resource challenges”. Coupled approaches answer some of the limits of the integrated modeling approach identified by Wiebe *et al*. [52], especially “a potential mismatch between the dynamics generated endogenously in the computer model and the narrative assumptions about exogenous factors” (see also [53]). To deal with these difficulties, Agrimonde-Terra made two methodological choices.

First, it decided to use a conceptually simple biomass balance model. Such models (also used in [9, 15, 16]) are different from market and trade economic models, such as those used in Integrated Assessment Models (IAMs). Market and trade economic models rely on a set of price-response parameters, which link quantity and price level adjustments and result in changes consistent with profitability patterns, a mechanism that is not present in biomass balance models. Despite this limit, we chose a biomass balance approach for tractability, simplicity and transparency reasons. Many variables are exogenous in a biomass balance model. This means it is easy to implement directly long-term scenarios in this type of tool (for example, direct shocks on food quantities of the various products). This is not the case with market and trade economic models, in which most of the variables are endogenous (especially in computable general equilibrium models), and the scenarios can be implemented only indirectly (for example, shocks on consumers’ income and preferences). In addition, at least for this specific study, a biomass balance model appeared to be a simpler and more transparent tool compared to an economic model, making discussions about the results and their main insights easier within the scenario committee, between economists and non-economists, and between scientists and other stakeholders.

Second, the morphological approach made it possible to systematically link the qualitative assumptions and their quantitative translation into simulation input data. The morphological analysis, in which all the assumptions used in each scenario are listed, provides a unified reasoning framework which enables the coupling with a quantified model. We have developed translation matrices that detail the translation from the qualitative assumptions to the values of input variables for the quantitative model.

This approach based on morphological analysis, biomass balance model and translation matrices responds to some of the issues identified in the scientific literature on land-use scenarios. First, Mallampalli *et al*. [54] identify a need to make the “translation step” that “relate[s] qualitative narrative scenarios to specific (…) input values” more “transparent, and reproducible” in order to improve the coherence between “narrative scenarios” and “quantitative assessments” of future land use. Translation matrices and morphological analysis help to increase transparency, to compare scenarios from different studies and so could contribute to improve the scientific debate [52]. Second, some authors acknowledge the need to explore a larger range of future land use in order to deal with higher uncertainty and with radical changes toward sustainable futures in land use [53, 55–56]. Our method combines both the possibility of a broad exploration of possible futures of land use provided by the morphological analysis and a simple and tractable model suited for simulating contrasting futures [38, 55]. As a result, the method proposed by Agrimonde-Terra could contribute to making the debate on systemic interactions inside land use and food security systems clearer and more transparent.

### A new set of alternative future diets

Diets are changing at a rapid pace as a result of multiple factors such as urbanization, rising incomes, lifestyle and eating habits, food supply chains, agriculture and nutrition policies [57]. Future diets could have dramatic impacts on land use, environment, climate change and health (for example, [25, 9, 4]). Therefore, the way we build future diet assumptions is of major importance in improving our anticipation of future issues. In a review of global food security scenarios, van Dijk and Meijerink [58] recommended the better inclusion of indicators for diet composition and content, in addition to energy intake, in order to assess nutrition security including all types of malnutrition.

One major historical change in diets, which occurred in the last century mainly in high-income countries, was the increase in the consumption of animal-based foods and the reduction in cereal consumption [59]. More recently (i.e., since 1990–2000) at the global level, diets have shifted towards an increased consumption of ultra-processed foods [60], which is strongly correlated with increased prevalence in NCD [61–62] such as type 2 diabetes [63], cardiovascular diseases [64], certain cancers [65] and in overweight and obesity [57]. Diets with a high proportion of ultra-processed foods exhibit higher contents of sugar, saturated and trans fats and refined cereals. They also provide lower protein, fibre and micronutrient contents than other diets and are unbalanced diets [66]. This situation is modifying our conception of global food and nutrition security [67].

In Agrimonde-Terra, future global diets in 2050 were mainly based on a trend analysis of dietary changes in different parts of the world. Four key features were emphasized. First, world regions still have large differences in terms of dietary composition [68] and the traditional diets that still exist in some low-income countries can offer good nutritional quality [69]. Second, urbanization processes and the resulting changes in incomes and lifestyle have encouraged the consumption of animal-based foods ([70]; see also [71] for the specific case of China). Urban food consumption is generally characterized by increased reliance on food services such as restaurants, street foods, snack products, ready-to-eat meals and soft drinks, and a reduced incidence of meals at home. Third, increased consumption of ultra-processed foods is linked to changes in food supply chains. In low and middle-income countries, large and small retailers and transnational food and logistics companies, using marketing and advertising, are currently reshaping food supply chains and food environments both in urban and rural areas [72–73]. Even in low-income countries, changes in food supply chains promote access to processed and ultra-processed low-priced foods and lead to a generalization of diets rich in ultra-processed foods [60]. Fourth, as they link rural and urban areas in food chains, intermediate cities are vectors for shaping rural economies and reinforcing food security in response to urban food demand [74–75]. Some cities or regions are addressing the reinforcement of regional food networks linking urban and rural areas to provide “nutritious, sustainable and equitable supply of food” [76–77].

**Fig 4:**
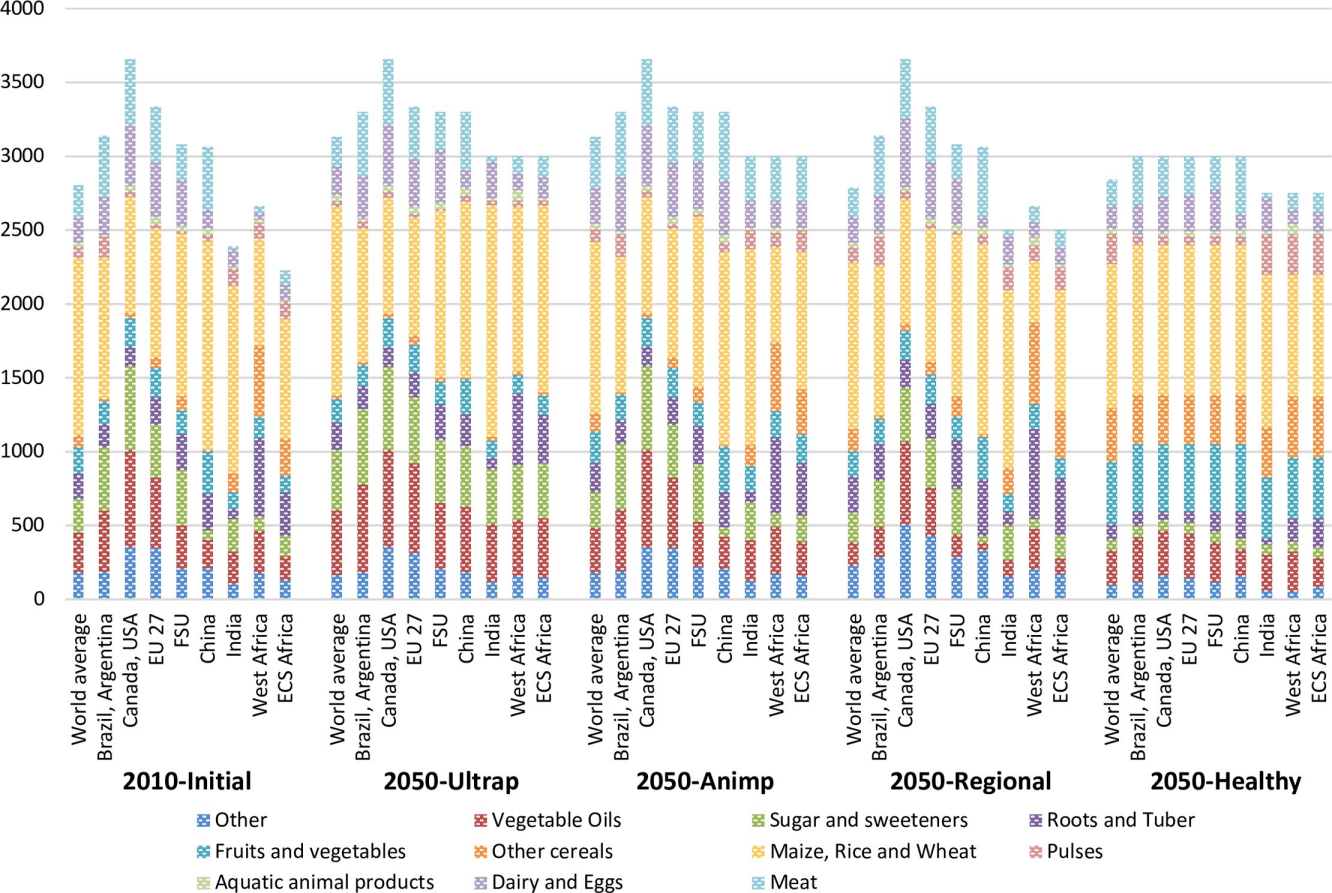
Food diets in 2010 and in 2050 under alternative diet assumptions at the world level and for selected world regions (kcal available/cap/day). Sources: Calculated from GlobAgri-AgT database. Each bar represents food available per food groups in kcal/cap/day at the world level (the first bar of each group) and for eight world regions: Brazil-Argentine; Canada-USA; European Union (UE 27); Former Soviet Union (FSU); China; India; West Africa; East, Central and South Africa (ECS Africa).

Based on these analyses, four assumptions for diets in 2050 (Fig 4) were built to explore the wide range of possible future diets and their potential impacts: a diet based on animal products, a diet based on ultra-processed foods, a diet based on (macro) regional traditional diets and a healthy diet. These diet change assumptions relate to different types of urbanization processes and urban-rural interactions. They also call for different nutrition and food as well as trade policies (for example, taxes on unhealthy foods, subsidized fresh products, support for school food programs and open-air markets; see [27]).

Here, we would like to focus on the healthy diet. The explicit aim of this diet, which is based on World Health Organization (WHO) dietary guidelines, is to reduce all forms of malnutrition (undernutrition, overweight and obesity and NCD), by diversifying food consumption and reducing the share of unhealthy foods in diets. As shown in Fig 4, the healthy diet exhibits a high share of fruits and vegetables, as well as pulses, and a low share of sugar, vegetable oils and animal-based foods as a result of the low consumption of ultra-processed foods. In terms of energy intakes, the healthy diet assumption strives for an improvement in the energy balance by reducing energy intakes in high- and middle-income countries and increasing them in low-income countries. Based on these principles, this diet has been adapted to the various world regions considering current diets and cultural traditions (Fig 4).

The healthy diet in Agrimonde-Terra is consistent with the recent healthy diet proposed by the EAT-Lancet Commission’s study [21], where the reference diet is characterized by a low intake of refined grains, processed meats, saturated fats, hydrogenated oils and sugar, a modest intake of meat, and a high intake of protein sourced from plants, and carbohydrates sourced from whole grains and fruits and vegetables. It should be noted that refined grains, processed meats, saturated fats, hydrogenated oils and added sugar are major components of ultra-processed foods.

This new generation of diet scenarios reflects a step forward in acknowledging the diverse forms of malnutrition. In comparison, traditional scenarios for sustainable diets have promoted reduced consumption of animal-based foods, replacing them with plant-based foods [25, 17, 18, 11]. For Milner and Green [78] such an option “might not be an equitable or ethical approach in low-income country settings where undernutrition remains prevalent”. In Agrimonde-Terra, the healthy diet combines two opposite strategies regarding the specific issue of food and nutrition security in each world region. In high- and middle-income countries, the target is to decrease animal food consumption, while in some low-income countries, where protein intakes are too low, the objective is to increase it, together with a partial substitution of animal protein with plant-based protein (mainly from pulses), in order to raise daily protein intakes. For instance, relative to the initial situation, our healthy diet involves a 39% reduction in the daily consumption of animal products in North America and a 35% increase in ECS Africa, together with a 130% increase in the daily consumption of pulses (Fig 4). More generally, the Agrimonde-Terra healthy diet considers that a diversification strategy for diets, including more diverse plant-based foods, could partly resolve undernutrition and child stunting in low-income countries by reducing micronutrient deficiencies [79].

Finally, we note that Agrimonde-Terra’s ‘Healthy’ scenario (involving the healthy diet) induces a limited expansion in world agricultural land area (Fig 3; see S1 Fig for regional impacts). This is coherent with results from Springmann *et al*. [80] who conclude that scenarios with “balanced energy intakes, and diets with low animal-source foods and in line with dietary guidelines” have the highest human health and environmental benefits.

### A new set of alternative future pathways for the global food system

#### Agrimonde-Terra scenarios: Similarities and differences with other sets of global scenarios

Although designed for different purposes and using an alternative method, Agrimonde-Terra’s five scenarios exhibit similarities with existing sets of global scenarios. Using the ‘scenario families’ categorization proposed by van Vuuren *et al*. [81], ‘Metropolization’ would likely belong to the ‘Economic optimism/Conventional markets’ family of scenarios (such as MEA’s Global Orchestration scenario, the A1 scenario among the SRES and the SSP5 Fossil-fueled development scenario from the SSPs). Similarly, ‘Healthy’ would belong to the ‘Global sustainable development’ family (sharing elements with the MEA TechnoGarden scenario, the SRES B1 scenario, the SSP1 Sustainability scenario, the Agrimonde 1 scenario and the Towards sustainability scenario from [26]). The ‘Communities’ scenario shares the idea of crisis and global fragmentation with the ‘Regional competition/Regional markets’ family of scenarios (involving the MEA’s Order from Strength scenario, the A2 scenario among the SRES and the SSP3 Regional Rivalry scenario). However, ‘Communities’ does not fit exactly with this scenario family since it involves an additional climate and ecological crisis, which makes the context far more challenging for world food systems. Finally, the ‘Regionalization’ and ‘Households’ scenarios are quite original and do not fit with any of the scenario families proposed by van Vuuren *et al*. [81].

As shown in Fig 3, comparing the impacts on land-use change of the Agrimonde-Terra and SSP scenarios confirms that there are similarities between ‘Metropolization’ and SSP5, ‘Healthy’ and SSP1 and, to a lesser extent, ‘Communities’ and SSP3. It also shows that ‘Regionalization’ and SSP2 both play the role of the median scenario in each set of scenarios, even if they involve radically different pathways for the global food system.

When comparing the impacts of scenarios on world cropland area (Fig 3, Panel A), we note that SSP5-RCP6.0 has rather similar impacts to ‘Metropolization_Ultrap’; SSP1-RCP2.6 has rather similar impacts to ‘Healthy_C’; SSP3-RCP6.0 has cropland impacts which can be considered similar to those of ‘Communities_AE’. However, our Animp variant of ‘Metropolization’ and Collapse variant of ‘Communities’, the scenarios which use the most cropland at the world level, induce cropland-use change impacts that are quite different from those of the SSPs. Both sets of scenarios use different quantitative assumptions (notably on population and climate change impacts) and different simulation models, so there are many reasons explaining their differentiated impacts on the world cropland area. However, we think that the two ‘extreme’ assumptions, involved in the Animp and Collapse variants, and which are not considered in the SSPs, can partly explain these differences. The Animp variant involves a very significant increase in animal-based food consumption all over the world and especially in developing regions, while the Collapse variant assumes an ecological crisis resulting in stagnation in crop yields all over the world (meaning decreasing crop yields when accounting for the impact of climate change) and lower improved performance of livestock systems. Furthermore, as we calibrated crop yields in 2050 based on differentiated levels of yield gap reduction, we implicitly assumed no advance in yield frontier and so adopted rather conservative yield assumptions.

Similarities between the Agrimonde-Terra and SSP scenarios are less obvious when comparing their impacts on the world pastureland area (Fig 3, Panel B). The outcomes of three Agrimonde-Terra scenarios are well above the whole range covered by the SSPs: ‘Metropolization_Animp’, ‘Regionalization_B’ and ‘Communities_Collapse’. Once again, there are many reasons explaining these differentiated impacts. But we think that one important reason lies in our feed-to-output ratios and system shares in production assumptions for the ruminant sectors, as well as our assumptions on changes in pasture productivity in 2050, which are rather conservative. Indeed, there is still a great uncertainty on both the production potential of current pastureland areas and the future development of pastures [82–83]. Another reason which strongly affects the impacts of scenarios on land-use changes is the assumed shift from ruminant to poultry meat in food diets: the larger this shift, the higher the increase in the cropland area required relative to pastureland area. All these reasons could explain why, despite a narrative close to SSP1-RCP2.6, ‘Healthy’ leads to divergent results: the world pastureland area increases with ‘Healthy’ while it decreases significantly with SSP1-RCP2.6. However, we think that in addition to the reasons mentioned above, ‘Healthy’ involves a food diet in 2050 that implies increased meat consumption in developing countries (so, sub-Saharan Africa).

#### The ‘Regionalization’ scenario: A third way between market globalization and market fragmentation

Despite sharing a reference to the regional level, ‘Regionalization’ does not fit with the ‘Regional competition/Regional markets’ family of scenarios. This family includes crisis scenarios where the world is fragmented with tensions among regions, high population, medium to low economic growth and little concern for environmental protection. ‘Regionalization’ could be more in line with the ‘Regional sustainable development’ family (including the MEA Adapting Mosaic scenario and the SRES B2 scenario). But, according to van Vuuren *et aI*. [81], scenarios in this family are usually badly quantified so that, based on the quantification, they are closer to business-as-usual scenarios (like the SSP2 Middle of the road scenario, for instance).

In Agrimonde-Terra, the ‘Regionalization’ scenario offers a third way between globalization and fragmentation, which is not a ‘Middle of the road’ or a ‘Business as usual’ pathway. Regarding the future of agriculture and food, the ‘Regionalization’ scenario explores a world where regional blocs shape food systems by promoting regional food culture and reconnecting the food industry to regional production through the development of medium-size cities and small towns. The new idea provided by this scenario is the realignment of food diets with supply systems at the macro-region level, with two interesting extensions. On the one hand, the ‘Regionalization’ scenario involves the location of agri-food industries and small-scale food processing in medium-size cities and small towns. This has a positive knock-on effect for rural development and contributes to decreased inequalities between urban and rural areas. On the other hand, the ‘Regionalization’ scenario reconnects crop and livestock production systems at the macro-region level. This contributes to reducing nitrogen and phosphorus imbalances at the world level [84–86].

However, as described earlier, the ‘Regionalization’ scenario may lead to significant agricultural land expansion at the world level and in some regions (mainly African ones; S1 Fig). This suggests that the ‘Regionalization’ scenario is not sustainable without significant improvements in crop and livestock productivity in some regions, especially in Africa. The discrepancy between the extent of world agricultural land expansion under both technology variants A and B of the ‘Regionalization’ scenario (Fig 3) provides a range of the sensitivity of its induced land-use impacts to the evolution in agricultural productivity around the world, and especially in regions where it is currently very low (S1 Fig).

#### The ‘Communities’ scenario: A perfect storm is still possible

In Agrimonde-Terra, the idea of crisis and global fragmentation is rather prominent in the ‘Communities’ scenario. However, ‘Communities’ does not fit with the ‘Regional competition/Regional markets’ family of scenarios because it envisages, in addition to financial, economic and geopolitical crises, a climate and ecological crisis leading to a downward spiral that could lead to the collapse of agricultural production systems. With this scenario and, contrary to most existing sets of scenarios, Agrimonde-Terra considers that a ‘perfect storm’ is still possible [87–88] and should be part of the debate.

In contrast to existing scenarios, ‘Communities’ explores a world where conventional production systems suffer from the feedback effects of climate warming and biodiversity loss, resulting in a less favorable context for the improvement of agricultural performance. In this context, the ‘Communities’ scenario considers two trajectories that could coexist. The collapse variant is totally absent from existing scenarios. It assumes that farmers fail to organize themselves and that the resulting subsistence farming based on conventional intensification generates collapse and stagnation or reduced improvements in crop and livestock system performance. The agroecology variant shares elements with scenarios involved in van Vuuren *et al*.’s [81] ‘Regional sustainable development’ family of scenarios. In this variant, farmers succeed in organizing themselves within their community to develop agroecological farms and local food and energy systems.

As previously shown, the agroecology variant of the ‘Communities’ scenario would make it possible to limit the expansion in world agricultural land. However, we should remind ourselves that this is at the cost of reduced energy content in food diets in most world regions. In contrast, the ‘Communities’ scenario with the collapse variant is our worst scenario regarding the expansion in agricultural land at the world level. Such a scenario would induce dramatic deforestation and resource degradation in all regions.

## Conclusion

Agrimonde-Terra’s exploratory scenarios offer some possible pathways to reach food and nutrition security and sustainable agriculture at the world level, but these pathways are narrow, with potential risks in terms of agricultural land expansion, malnutrition and food insecurity.

Compared to other sets of global scenarios, Agrimonde-Terra places more emphasis on four dimensions and invites future foresight analyses of global food systems to further explore them. It proposes a methodological framework that combines an explorative qualitative approach for dealing with increasing uncertainty and complexity, and a tractable and simple quantitative model suited to simulating highly contrasting scenarios. Such a method helps to improve transparency and coherence between scenario narratives and quantitative assessment. Agrimonde-Terra scenarios comprise a wide range of alternative diets, with contrasting underlying nutritional and health issues, which accompany contrasting urbanization and rural transformation processes. The insights provided by scenarios in terms of land use and food and nutrition security show the importance of considering strongly contrasting dietary patterns and highlight the key role of rural-urban relationships in the transformation of food value chains. Results confirm that there are win-win scenarios where changing global diets towards healthier patterns can help to limit the expansion in agricultural land area. Agrimonde-Terra scenarios share some similarities with existing sets of global scenarios, notably the SSPs, but are most often less optimistic with regard to agricultural land expansion up to 2050. Specifically, Agrimonde-Terra’s scenarios use more pastureland, for two possible reasons: firstly because its assumptions on the productivity increases in both pasture and ruminant livestock systems are rather conservative, and secondly because it is assumed that even a healthy diet would involve increased meat consumption per capita in some developing world regions. Such results call for further research on the production potential of both pastureland areas and future livestock systems all over the world. Agrimonde-Terra’s scenarios enlarge the scope of possible futures by proposing two pathways uncommon in other sets of global scenarios. The first proposes to explore possible reconnection of the food industry and regional production, within supranational regional blocs. The second means that we should consider that a ‘perfect storm’, induced by climate change and an ecological crisis combined with social and economic crises, is still possible, and should be part of the debate, as suggested by the IPCC special report on climate change and land [89].

The current Covid-19 pandemic clearly shows that scenario studies should enlarge the scope of possible futures. In this regard, it is worth noting that both Agrimonde-Terra scenarios, which are original relative to other sets of global scenarios, describe changes and raise issues that resonate with observed and expected impacts of this pandemic on food systems and food security. Firstly, the current context corresponds to the multi-crises context and induced world food security concerns as described in the Communities scenario. Secondly, the revealed fragility of global supply chains facing a pandemic with a lockdown response calls for improved resilience of vital chains such as food supply chains. The regionalization of food systems could be an answer and this is the pathway involved in the Regionalization scenario.

Agrimonde-Terra’s scenarios were built to contribute to the ongoing debates on land-use trajectories, to facilitate informed decision-making and to help identify new research questions. Therefore, they can be used by various actors, from decision makers to the scientific community, at various scales, from global to national or even infra-national. In this regard, Agrimonde-Terra’s outputs have already been used in Tunisia, with a group of farmers, members of Ministries and researchers to build land-use scenarios at the national scale in order to reflect on strategic options for Tunisian agriculture [90]. In India, Agrimonde-Terra’s trend analysis, assumptions on future changes in drivers and scenarios were the support of a scientific seminar with researchers from INRA, CIRAD and the Indian Council of Agricultural Research with the aim of identifying new research project areas. There are also current opportunities for Agrimonde-Terra’s outputs (method, simulation model and driver assumptions) to be involved in research projects on the future of European cropping and livestock systems. Agrimonde-Terra’s outputs can be a tool for assessing future changes in food systems providing a large range of possible futures and their quantification, and for identifying the most important challenges that we should anticipate for building sustainable and healthy pathways for food systems.

## Supporting information

S1 File The GlobAgri-AgT model. (DOCX)

S2 File Translation matrices of the drivers of Agrimonde-Terra. (DOCX)

S1 Table Consequences of the Agrimonde-Terra scenarios on land use, food security and nutrition. (DOCX)

S1 Fig Regional agricultural land area in the initial 2010 situation and in 2050 in the various scenarios. (DOCX)
