# Supplementary material for: Exploring the future of land use and food security: A new set of global scenarios
Source: PLoS One. 2020 Jul 8;15(7):e0235597. doi: 10.1371/journal.pone.0235597 (PMC7343151; doi:10.1371/journal.pone.0235597)
Supplement: S1 File — (DOCX) [file pone.0235597.s001.docx]

**S1 File. The GlobAgri-AgT model**

This section relies on chapter 3 (Globagri-AgT database and model) in Le Mouël et al. (2018).

**Model structure**

The GlobAgri-AgT model considers 38 agri-food products (Table 1) and 14 world regions (Table 2).

For each product *i* ($i\in I)$ in each region *j*, at each period *t*, there is a resource-utilization balance equation: ${Prod}_{ijt}+{Imp}_{ijt}-{Exp}_{ijt}={Food}_{ijt}+{Feed}_{ijt}+{Oth}_{ijt}+{Waste}_{ijt}+{VStock}_{ijt}$

Where *Prod* is the domestic production, *Imp* imports, *Exp* exports, *Food* the domestic food consumption, *Feed* the domestic feed use, *Oth* the other domestic uses, *Waste* the waste and *VStock* the stock change.

For all vegetal products ($v\in I$), domestic production equals harvested area (*A*) multiplied by per-hectare yield (*Y*): ${Prod}_{vjt}=A_{vjt}*Y_{vjt}$

For all products, the domestic feed use is a linear function of the domestic production of reference animal products ($a\in I$): ${Feed}_{ijt}=\sum_{a} \beta_{iajt}*{Prod}_{ajt}$

Where $\beta_{iajt}$ is the fixed transformation coefficient of product *i* into animal product *a* in region *j* for year *t* ($\beta_{iajt}$ are also called the feed-to-output ratios). For each animal product, they are a weighted average of the corresponding feed-to-output ratios observed in the various production systems co-existing in the sector concerned. For the five sectors under consideration (dairy, beef, small ruminants, pork and poultry), the various production systems are those suggested by Herrero *et al*. (2013): mixed, pastoral, urban and other systems for ruminant sectors and urban and other for non-ruminant sectors.

For all products *i*, imports are a fixed share of total domestic use:

$${Imp}_{ijt}=\alpha_{ijt}*({Food}_{ijt}+{Feed}_{ijt}+{Oth}_{ijt}{+Waste}_{ijt}+{VStock}_{ijt})$$

Where $\alpha_{ijt}$ is the import dependence coefficient of region *j* for product *i* in year *t*.

Exports of product *i* by region *j* are a fixed share of the world market size of product *i*:

${Exp}_{ijt}=\sigma_{ijt}*(\sum_{j} {Imp}_{ijt})$

Where $\sigma_{ijt}$ is the world export market share of region *j* for product *i* in year *t*.

Import and export specifications in GlobAgri-AgT imply some rigidity in international trade. However, import dependence coefficients ($\alpha_{ijt}$) and/or world export market shares ($\sigma_{ijt}$) may be changed exogenously as part of simulated scenarios (e.g. the ‘Regionalization’ scenario).

The model is closed firstly adding a world trade equilibrium equation for each product:

$\sum_{j} {Imp}_{ijt}=\sum_{j} {Exp}_{ijt}$

and secondly adding an agricultural land constraint equation in each region: $\sum_{v} {Surf}_{vjt}\leq\bar{{Surf}_{jt}}$

This agricultural land constraint may be defined for various sets of products *v* so that the *Surf* and $\bar{Surf}$ may have different meanings: cropland, pastureland or total agricultural land area for instance. In GlobAgri-AgT, because of the lack of data regarding the maximum pastureland area in each region, we defined the agricultural land constraint on the cropland area. Hence, ${Surf}_{vjt}$ is the cultivated area devoted to crop product *v* in region *j* during year *t* and $\bar{{Surf}_{jt}}$ is the maximum cultivable area in region *j* in year *t*. In other words, we assume that in each region *j*, cropland is constrained by the maximum cultivable area while pastureland may adjust freely to all the shocks introduced into the model.

Finally, an additional equation is linking the total harvested area to the total cultivated area in each region: $\sum_{v} {Surf}_{vjt}=e_{jt}*(\sum_{v} A_{vjt})$

Where $e_{jt}$ measures the level of cropping intensity in region *j* for year *t*. This ratio is lower (greater) than one when the cultivated area is lower (greater) than the harvested area, indicating the extent of multi-cropping (the extent of fallow land or of harvest abandonment) in the concerned region.

**Model solving**

Starting from the initial 2007/09 situation, where domestic resources-utilizations and world trade are balanced for all products and the observed cropland area is lower or nearly equal to the maximum cultivable area in all regions, let’s assume that food consumption of product *i* increases in region *j*.

According to GlobAgri-AgT specification, region *j* covers these additional needs partly by rising imports and partly by expanding domestic production. This results in an expansion of cropland and, possibly, pastureland areas in region *j*:

- If region *j*’s cropland area remains lower than region *j*’s maximum cultivable area, then the resolution of the model stops;
- If region *j*’s cropland area becomes greater than region *j*’s maximum cultivable area, then two stages are considered:

1. region *j*’s exports are first evenly reduced (through equi-proportional decrease in its world export market shares $\sigma_{ijt}$ ) until the domestic cropland area falls below the maximum cultivable area. At this stage, the resolution of the model stops;
2. if, even with zero exports, region *j* still needs more cropland area than its maximum cultivable area, then region *j* starts increasing its imports (through increases in import dependence coefficients $\alpha_{ijt}$). In other words, region *j* increases the share of its food needs, which is covered by imports in order to reduce the required rise in domestic production and save some cropland area.

Therefore, in this last case, the world export market shares and import dependence coefficients of land-constrained regions become endogenous.

**Table 1. Agri-food aggregates in GlobAgri-AgT**

| Aquatic animals  Bovine meat  Dairy  Eggs  Pork meat  Poultry meat  Small ruminant meat | Fibres etc.  Fruit and vegetables  Pulses  Roots and tubers  Maize  Other cereals  Rice  Wheat  Sugar plants and products  Other products | Other oilcrops  Cake other oilcrops  Oil other oilcrops  Oilpalm fruit  Palm product oil  Palm kernel cake  Rape and mustard seeds  Rape and mustard cake  Rape and mustard oil  Soyabeans  Soyabean cake  Soyabean oil  Sunflower seeds  Sunflower seed cake  Sunflower seed oil | Grass (grass from direct grazing and as silage of permanent pastures)  Grass-like forages (mixed grass and ryegrass from temporary pastures)  Other forages (alfalfa and fodder crops: beats, vegetables, sorghum, maize etc.)  Occasional feeds (food leftovers, cut-and-carry forages and legumes, roadside grasses)  Stover (crop residues) |
| --- | --- | --- | --- |

**Table 2. Broad geographic regions in GlobAgri-AgT**

| Brazil/Argentina  Rest of America  Rest of the world | Canada/USA  EU-27  Oceania  Former Soviet Union | China  India  Rest of Asia  Near and Middle East | North Africa  West Africa  East, Central and Southern (ECS) Africa |
| --- | --- | --- | --- |

**References**

Herrero M, Havlik P, Valin H, Notenbaret A, Rufino MC, Thornton PK, Blümmel M, Weiss F, Grace D, Obersteiner M. Biomass use, production, feed efficiencies, and greenhouse gas emissions from global livestock systems. PNAS 2013; 110(52): 20 888-20 893.
