# Supplementary material for: Exploring the future of land use and food security: A new set of global scenarios
Source: PLoS One. 2020 Jul 8;15(7):e0235597. doi: 10.1371/journal.pone.0235597 (PMC7343151; doi:10.1371/journal.pone.0235597)
Supplement: S2 File — (DOCX) [file pone.0235597.s002.docx]

**S2 File. Translation matrices of the drivers of Agrimonde-Terra**

This section relies on chapter 14 (Land-use change impacts of Agrimonde-Terra scenarios: An assessment with the GlobAgri-AgT model) in Le Mouël et al. (2018).

**Global Context**

The five pathways for the global context are: i) Sustainable and cooperative world; ii) Regionalization and energy transition; iii) Economic and political fragmentation; iv) Conventional development led by market forces; and v) Non-State actors.

The general rules for building the quantitative assumptions on population change on the one hand and on trade coefficients on the other hand are the following:

- Changes in total world and regional population are the same in all pathways. The median projection up to 2050 provided by the United Nations (2015 revision) is used.
- Import coefficients of regions are exogenously reduced in the ‘Regionalization and energy transition’ future pathway in order to figure the inter-regional trade impact of the supranational regional blocks development as well as the implementation of the ‘food sovereignty and subsidiarity’ principle. From 2010 to 2050, import coefficients for all products are equally reduced by -25% in all regions except the Near and Middle East and North Africa (both regions being constrained by their cultivable land). Additional reductions are implemented as to decrease the net import dependence of net importing regions until they have not reached their maximum cultivable area.^^[[1]](#footnote-1)^^ Regional import coefficients are exogenously unchanged in all other 4 global context pathways.
- World export market share coefficients are not changed exogenously whatever the global context pathway.

**Climate change and mitigation**

Climate change patterns to 2050 are described through three pathways, inspired from the Representation Concentration Pathways (RCP) of the fifth assessment report of the IPCC (Intergovernmental Panel on Climate Change): i) Stabilisation of global warming (close to RCP 2.6); ii) Moderate warming and ii) Runaway climate change (close to RCP 8.5).

The general rules for building the quantitative assumptions on concerned entry variables of the model are as follows:

- We assume that in the 2010 initial situation the maximum cultivable area (i.e., the maximum area which can be devoted to arable and permanent crops) in each region equals the area under suitability indices 1 to 4 according to the GAEZ approach. In the Global Agro-Ecological Zones (GAEZ) approach, land is classified according to its quality or suitability for agricultural production. There are eight classes ranging from ‘very suitable’ to ‘not suitable’. GAEZ suitability indices 1 to 4 correspond to ‘very suitable’ to ‘moderately suitable’ land.^^[[2]](#footnote-2)^^
- We assume that up to 2050 this maximum cultivable area is affected by climate change. To quantify the climate change effects we use Zabel et al. (2014)’s results^^[[3]](#footnote-3)^^ and adopt the following assumptions: i) no change in the ‘Stabilization of global warming’ pathway; ii) change according to Zabel et al.’s results in the ‘Runaway climate change’ pathway; iii) half the change according to Zabel et al.’s results in the ‘Moderate warming’ pathway.
- We assume that climate change is likely to affect the evolution of crop yields induced by changes in cropping systems up to 2050. For quantifying the climate change effects on crop yield evolution we use Müller and Robertson (2014)’s results and adopt the following hypotheses: i) no change in the ‘Stabilization of global warming’ pathway; ii) change according to Müller and Robertson’s results in the ‘Runaway climate change’ pathway; iii) half the change according to Müller et Robertson’s results in the ‘Moderate warming’ pathway.
- Due to data uncertainties and absence of consensus in the literature we did not establish quantitative hypotheses on the impact of climate change and mitigation pathways on grass and forage yield change, nor on livestock productivity change.
- Quantitative mitigation hypotheses (Table 1) have been established based on IPCC work (IPCC, 2014). In the ‘Stabilization of global warming’ pathway and in the ‘Runaway climate change’ pathway, we assume that up to 2050 food, feed and energy crops are competing on the maximum cultivable area in each region: regional maximum areas available for food and feed crops equal regional maximum cultivable areas minus areas devoted to energy crops. In the ‘Moderate warming’ pathway, we assume that energy crops do not directly compete for cultivable land with food and feed crops since they are grown on abandoned land or on forest land.

**Table 1. World production of energy from biomass (EJ) in 2050**

|  | Energy crops (2G) | Forest | Residues | Other (algae) |
| --- | --- | --- | --- | --- |
| Runaway climate change | 30 | 0 | 30 | 0 |
| Stabilization of global warming | 30 | 30 | 30 | 12 |
| Moderate warning  Forest variant  Energy crops variant | 20  60 | 70  30 | 60  60 | 0  0 |

**Food diets**

Agrimonde-Terra built 4 hypotheses for the future of food diets up to 2050: i) Transition to diets based on ultra-processed products and transnational value chain (Ultrap); ii) Transition to diets based on animal products and urban style of life (Animp); iii) Regional diversity of diets and food systems (Regional) and iv) Food diversity for healthy dietary practices (Healthy).

The general rules that we established for building our quantitative hypotheses relate to both the change in the daily calories availability per capita and the share of the various groups of food in the diet. They are reported in Table 2.

**Table 2. General rules for the changes in food diets over 2010-2050 under the different pathways**

|  | **Ultrap** | **Animp** | **Regional** | **Healthy** |
| --- | --- | --- | --- | --- |
| Diet energy (Daily calories available per capita) | - Regions over 3300 kcal/cap/day in 2010: unchanged up to 2050  - Regions between 3000 and 3300 kcal/cap/day in 2010: increase to 3300 in 2050  - Regions under 3000 kcal/cap/day in 2010: increase to 3000 kcal/cap/day in 2050 | | - Unchanged relative to 2010 in all regions, except India and ECS Africa: increase up to 2500 kcal/cap/day in 2050  - When used with the “Communities” scenario: -10% reduction in all regions except India and ECS Africa | - Regions over 3000 kcal/cap/day in 2010: decrease to 3000 - Regions under 2750 kcal/cap/day in 2010: increase to 2750 - Regions between 2750 and 3000 kcal/cap/day in 2010: unchanged |
| Diet pattern | - Change according to 1998/2008 trends in Brazil. In all regions except Canada/USA: no change relative to 2010 - 2 diet share minimum thresholds: 13.5% for vegetable oils; 10% for animal products  - Within the meat group: strong substitution from ruminant meat to poultry meat | - In developed regions, unchanged relative to 2010 - In emerging regions, change according to 1998/2008 average trends in developed regions  - In developing regions, change according to 1998/2008 average trends in emerging regions  - Within the meat group: substitution from ruminant meat to poultry meat | - In each region, change according to the pattern observed in the region in 1970/72  - Diet share of animal products: 10% minimum threshold | - Diet share of animal products and pulses: 20% - Diet share of cereals: 50%, coarse grains accounting for ¼ to 1/3  - Diet share of fruits and vegetables: 15% - Diet share maximum thresholds: 10% for vegetable oils, 2.5% for sugar and sweetener  - Within the meat group: substitution from ruminant meat to poultry meat |

As shown in Fig 1, our assumptions induce very different changes in food diets from the reference year 2007-2009 (named ‘2010’ for simplicity) to 2050 across regions. For developed regions, such as Canada/USA, only the ‘Healthy’ pathway leads to significant change in food diets from 2010 to 2050. While all pathways imply moderate changes in food diets for emerging countries such as China. In contrast our assumptions induce significant changes in food diets, whatever the pathway, in developing countries such as ECS Africa. In all these regions, the Animp pathway implies a marked rise in the share of animal products, particularly of meat. The ‘Regional’ pathway is the one inducing the lowest changes.

It is worth noting that for ECS Africa, according to our assumptions:

- All pathways involve an increase in the daily calories availability per capita. As a sharp increase in population is also expected in ECS Africa, this means that food consumption will increase significantly under all pathways in this region (the same is observed for India).
- All pathways result in a rise in the share of animal products in diets. Once again, joint with the expected population increase, this rising share of animal products in diets will lead to huge increase in food consumption of meat, dairy and eggs under all food diet pathways in ECS Africa (even under the healthy diet assumption) (the same is observed in India and West Africa).

**Fig 1. Food diets in 2010 and in 2050 under the different food diets pathways in various world regions**

**
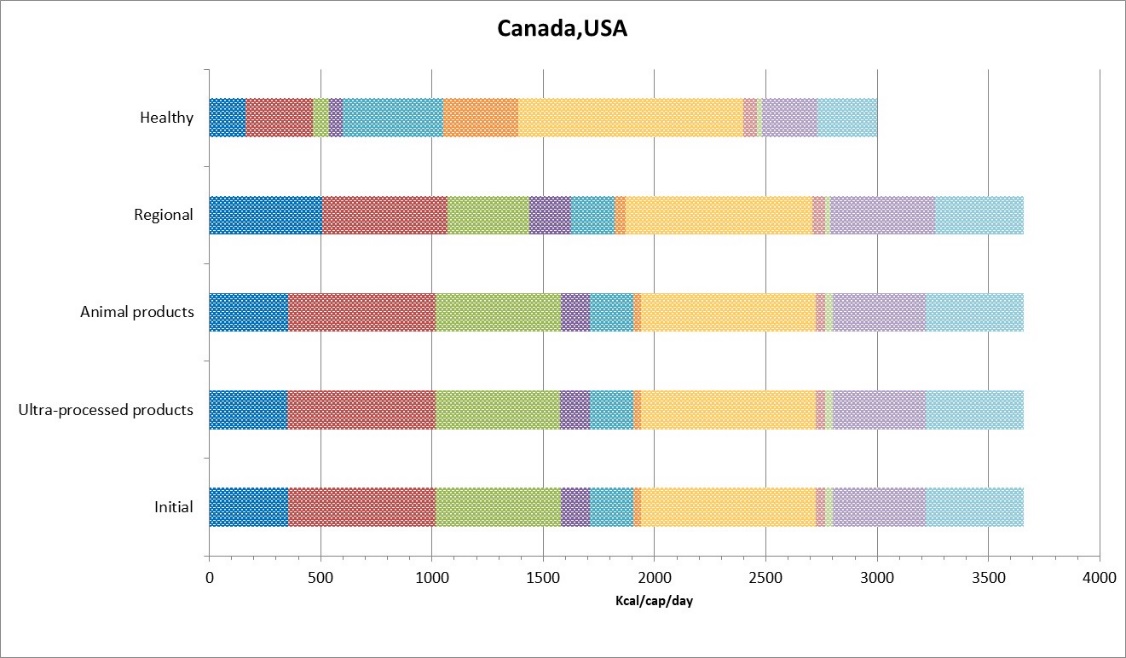
**

**
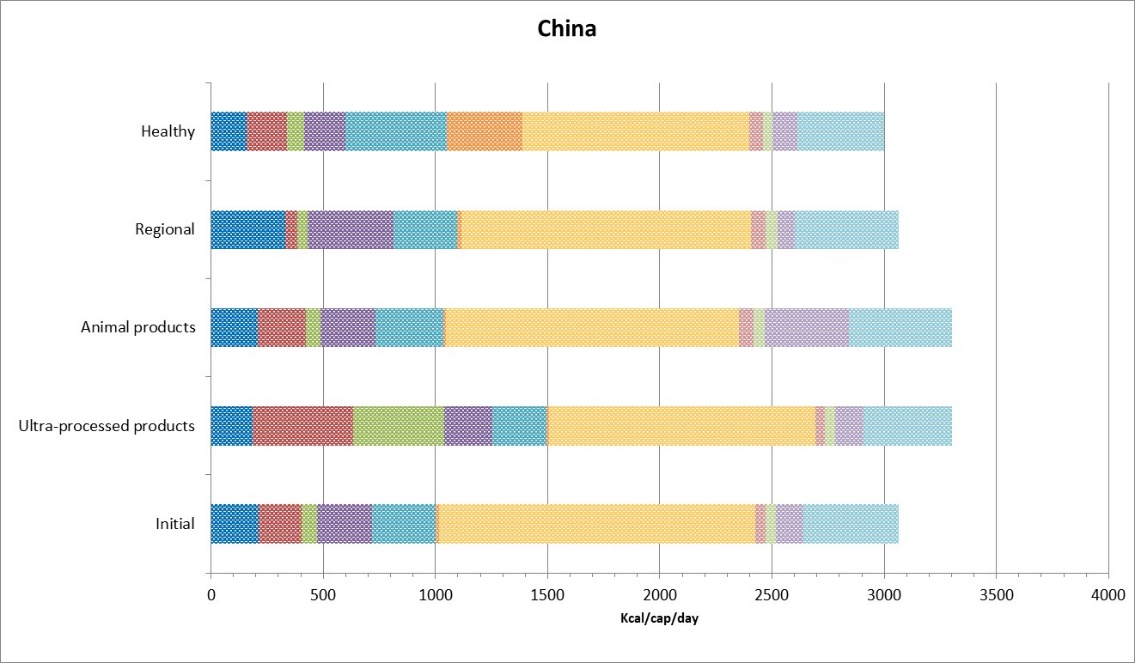
**

**
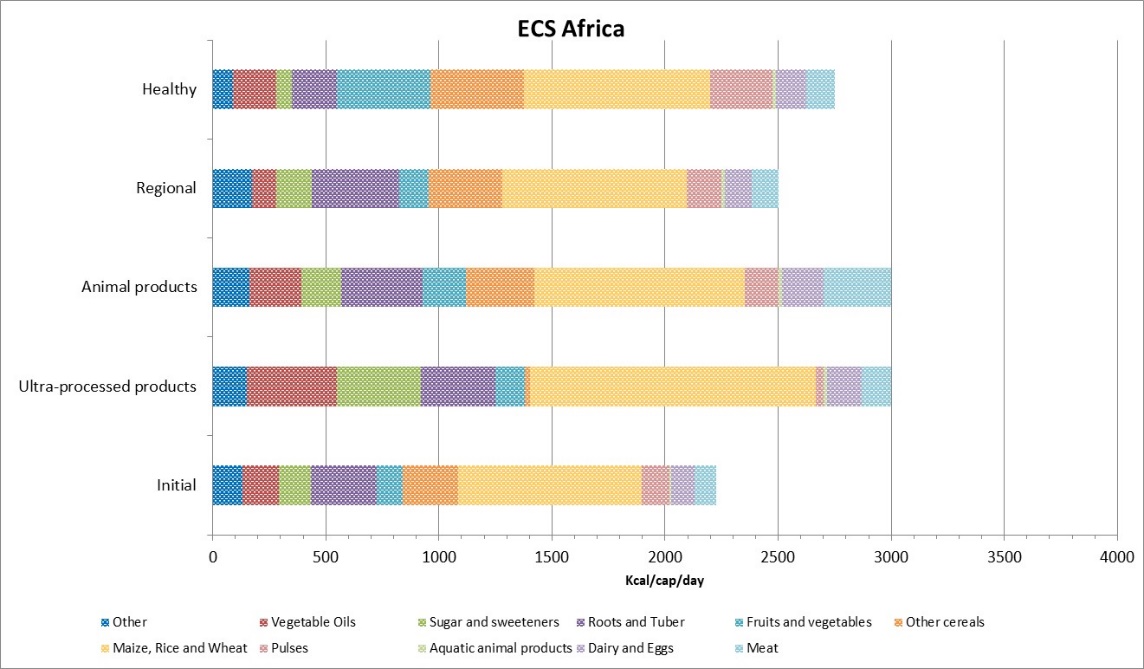
**

**Rural-Urban relationships**

The hypotheses for the future of rural-urban relationships up to 2050 have not been translated into quantitative hypotheses. In addition to the food consumption variable through food diet change, there are two entry variables of the GlobAgri-AgT model which are potentially influenced by changes in rural-urban relationships: the maximum cultivable area and the maximum pastureland area. Indeed, both areas may expand or decrease according to changes in rural-urban relationships. Unfortunately, we did not find enough empirical evidence about the impact of rural-urban relationships on land available to agriculture and we were not able to establish sound quantitative hypotheses for this driver of our “land use and food security” system.

**Farm structures**

The GlobAgri-AgT model has no entry variables allowing to deal with farm structures. Hence, the hypotheses about the future of farm structures up to 2050 have not been translated into quantitative hypotheses.

**Cropping systems**

Four distinct pathways for the evolution of cropping systems areconsidered: i) Conventional intensification; ii) Sustainable intensification; iii) Agroecology and iv) Collapse of cropping systems.

The general rules that we established for building our quantitative hypotheses relate to both the per-hectare yields and the cropping intensity ratios. They are reported in Table 3.

We adopted three general rules in order to translate the cropping systems pathways into quantitative assumptions for regional per-hectare yield changes:

- We calculated current yield gaps for Agrimonde-Terra’s crops and regions using data provided by the GAEZ database portal.^^[[4]](#footnote-4)^^ Then we assumed a level of yield gap reduction between 2010 and 2050, which is differentiated according to cropping pathways, and which we applied uniformly to groups of crops (cereals, protein seeds and other crops) and grass and forages (Table 3).
- We hypothesized that there is induced technical change together with induced change in the distribution of crops across land fertility classes, which results in greater rate of yield increase for crops which are the most demanded at the world level. Let us consider a group of crops, say cereals, the above described yield gap reduction applies to the average cereal yield. Then we assume that within the cereal group, cereal crops which are most demanded will benefit of above-average yield gap reductions (H in Table 3), while cereal crops which are less demanded will experience below-average yield gap reductions (L in Table 3). The retained indicator for “most demanded/less demanded” crops is the change in the respective crop shares in the average world diet under the various food diet pathways
- Grass and forage crops benefit from the same yield gap reduction than other crop groups.

As far as cropping intensity ratios are concerned, we adopted the following general rules:

- Based on existing literature and expert knowledge, it was not possible to differentiate the impacts of the various cropping pathways on cropping intensity ratios. Hence, we consider that all pathways have the same impacts on these ratios.
- We used Ray and Foley (2013)’s results in order to get a first assessment of potential changes in regional cropping intensity ratios up to 2050. However, as Ray and Foley’s work only considers the potential change in temperature, we revised this first assessment, taking into account the potential change in water availability for agriculture in the various regions as well as expert knowledge.

Finally, we assumed that in a low economic growth context, less budget resources are devoted to R&D so that the rate of technical change is lower. This is why when the ‘Agroecology’ pathway is used in the ‘Communities’ scenario (which involves a sluggish economic growth), the applied yield gap reductions and changes in cropping intensity ratios are less favourable.

**Table 3. General rules for translating the various cropping systems pathways into quantitative assumptions**

|  | **Average yield gap reduction** | **Higher (H)/Lower (L) yield gap reduction for specific crops** |
| --- | --- | --- |
| **“Conventional intensification”**  *combined Ttransition-Ultraprocessed*    *combined with Transition- Animp* | **-50%** | Grains: H maize/L coarse grains  Oilseeds and pulses: L pulses  Other products: H sugar crops  Same as above +  Grass and forage: H |
| **“Sustainable intensification”**  *combined with Healthy*  *combined with Regional* | **-40%** | Grains: H coarse grains/L others  Oilseeds and pulses: H pulses/L others  Other products: H fruits and vegetables/ L Others  Grass and forage: 0  Grains: H coarse grains, maize/L others  Oilseeds and pulses: H pulses, other oilcrops, soybean/L others  Other products: H roots and tubbers/ L Others  Grass and forage: 0 |
| **“Agroecology”**  *Combined with high economic growth*  *Combined with low economic growth* | **-30%**  **-15%** |  |
| *combined with Healthy*  *combined with Regional* |  | Grains: H coarse grains/L others  Oilseeds and pulses: H pulses/L others  Other products: H fruits and vegetables/ L Others  Grass and forage: 0  Grains: H coarse grains, maize/L others  Oilseeds and pulses: H pulses, other oilcrops, soybean/L others  Other products: H roots and tuber/ L Others  Grass and forage: none |
| **“Collapse”** | **0%** |  |

**Livestock systems**

Four hypotheses for the future of livestock systems were produced: i) Conventional intensive livestock with imported resources or with local resources (sometimes named Intensive and Intensive-local respectively); ii) Agro-ecological livestock on land in synergy with agriculture or urbanisation (sometimes named Agroecology); iii) Livestock on marginal land (sometimes named Rationalization) and iv) Backyard livestock (sometimes named Backyard).

In GlobAgri-AgT, regional livestock systems are quantitatively described and modeled based on data from Herrero et al. (2013). In each region, five livestock sectors are considered (dairy, beef, small ruminants, pork and poultry); they produce six animal products (milk and dairy, beef meat, small ruminant meat, pork meat, poultry meat and eggs). Each ruminant livestock sector comprises four production systems (Herrero et al.’s so-called mixed, pastoral, urban and other systems). Each monogastric sector involves two production systems (Herrero et al.’s so-called urban and other systems).

We faced some difficulties to quantify the hypotheses for the future of livestock systems and were forced to adopt some restrictive assumptions. Our difficulties resulted from, at least, two main reasons. First of all, only two entry variables of the GlobAgri-AgT model were available to quantify the hypotheses for the future while the latter involved livestock pathways that are differentiated on a set of various dimensions. Secondly, as far as ruminant sectors are concerned, there was not a clear ranking of the different production systems, from the least to the most intensive, emerging from the initial data we used. Hence, it was difficult to choose which production system(s) would expand more than the others in each retained hypothesis for the future (see below).

The two concerned entry variables of the GlobAgri-AgT model are: regional feed-to-output ratios (measuring the quantity of dry matter feed per unit of output produced) of each system in each sector; regional shares of the different production systems in the total output production of the considered sectors. Therefore, in GlobAgri-AgT, the overall productivity of a livestock sector (as measured by its global feed-to-output ratio) in one region may change between 2010 and 2050 through both the change in the feed-to-output ratios of the various systems in the sector (measuring the mixed effects of changes in the productivity per animal, in animal diseases and mortality and in the efficiency of feed rations) and the change in the relative shares in production of these various systems.

The general rules adopted for quantifying the livestock systems pathway of change are the following. They apply at the regional level:

- Each future pathway is associated with one or two specific production systems per sector: the chosen systems are those where changes are occurring, other systems remain constant over 2010-2050. For each livestock systems pathway, production systems concerned by changes are chosen as those fitting the best the dynamics involved in the pathway (Table 4).

- For production systems experiencing changes between 2010 and 20150, feed-to-output ratios are assumed to change according to projections to 2030 provided by Bouwman et al. (2005). This rule applies to all regions except West Africa and ECS Africa. In these both regions, we assume that feed-to-output ratios change two times faster than expected in Bouwman et al.’s projections for the beef sector.

- For all production systems, shares in the total production of concerned sectors are increased or decreased, depending on the pathway of change (Table 4).

- In the ‘Conventional intensive livestock with local resources’ pathway, the composition of feed rations of mixed systems in ruminant sectors and of urban systems in monogastric sectors are changed so that imported feedstuffs are replaced by locally produced feedstuffs. A locality indicator is first defined as to select the local feedstuffs. Then, within the energy-rich feed group and within the protein-rich feed group, local feedstuffs replace imported feedstuffs provided the energy content and the dry matter content of the ration remain unchanged. The balance of final rations in terms of protein is checked ex-post.

**Table 4. General rules for translating the various livestock systems pathways into quantitative assumptions**

|  | Conventional intensive livestock | Agroecological livestock | Livestock on marginal land | Backyard livestock |
| --- | --- | --- | --- | --- |
| Change in feed-to-output ratios* | | | | |
| **Ruminant**  Mixed  Pastoral  Urban  Other  **Monogastric**  Urban  Other | Decrease (Bouwman et al.)  Decrease (Bouwman et al.)  No change  No change  Decrease (Bouwman et al.)  No change | Decrease (Bouwman et al.)  Decrease, (Bouwman et al.)  No change  No change  No change  No change | Decrease (Bouwman et al.)  No change  No change  No change  Decrease (Bouwman et al.)  No change | No change  No change  No change  No change  No change  No change |
| Changes in production shares | | | | |
| **Ruminant**  Mixed  Pastoral  Urban  Other  **Monogastric**  Urban  Other | Increase  Decrease  Decrease  Decrease  Increase  Decrease | Increase  No change  Decrease  Decrease  Decrease  Increase | Decrease  Increase  Decrease  Decrease  Increase  Decrease | No change  No change  No change  No change  No change  No change |

* For feed-to-output ratios, decrease means that 1 tonne of animal product requires less quantity of dry-matter feed, implying higher productivity of the production system.

**References**

Bouwman AF, Van der Hoek KW, Eickhout B, Soenario I. Exploring changes in world ruminant production systems. Agricultural Systems 2005; 84: 121–153.

Herrero M, Havlik P, Valin H, Notenbaret A, Rufino MC, Thornton PK, Blümmel M, Weiss F, Grace D, Obersteiner M. Biomass use, production, feed efficiencies, and greenhouse gas emissions from global livestock systems. PNAS 2013; 110(52): 20 888-20 893.

IPCC. Climate Change 2014: Mitigation of Climate Change. Contribution of Working Group III to the Fifth Assessment Report of the Intergovernmental Panel on Climate Change [Edenhofer, O., R. Pichs-Madruga, Y. Sokona, E. Farahani, S. Kadner, K. Seyboth, A. Adler, I. Baum, S. Brunner, P. Eickemeier, B. Kriemann, J. Savolainen, S. Schlömer, C. von Stechow, T. Zwickel and J.C. Minx (eds.)]. Cambridge University Press, Cambridge, United Kingdom and New York, NY, USA; 2014.

Müller C, Robertson RD. Projecting future crop productivity for global economic modeling. Agricultural Economics 2014; 45: 37–50.

Ray DK, Foley JA. Increasing global crop harvest frequency: recent trends and future directions. Environmental Research Letters 2013; 8(4):44-41.

Zabel F, Putzenlechner B, Mauser W. Global agricultural land resources – A high resolution suitability evaluation and its perspectives until 2100 under climate change conditions. PLoS ONE 2014; 9(9): e107522. doi:10.1371/journal.pone.0107522.

1. The net import dependence of one region is the share of total net imports (imports-exports) in total domestic use, in kilocalories. For net importing regions with remaining cultivable land, additional reductions of import coefficients were applied: in each concerned region, all import coefficients were equally reduced, with the reduction level calibrated so as the net import dependence of the region is as close as possible to zero. These additional reductions concerned: East, Central and South (ECS) Africa, the Rest of America, China and the European Union (EU 27). [↑](#footnote-ref-1)
2. For more details, see <http://www.fao.org/nr/gaez/>. [↑](#footnote-ref-2)
3. More specifically we use updated results kindly provided by Florian Zabel, related to IPCC RCP 2.6 and RCP 8.5. [↑](#footnote-ref-3)
4. We used the data for year 2000. We retained the potential yields obtained with so-called “high inputs” cropping systems and, for both actual and potential yields, a weighted average of rainfed and irrigated yields (the weights being the relative shares of rainfed and irrigated land areas). [↑](#footnote-ref-4)
