# Supplementary material for: Exploring the future of land use and food security: A new set of global scenarios
Source: PLoS One. 2020 Jul 8;15(7):e0235597. doi: 10.1371/journal.pone.0235597 (PMC7343151; doi:10.1371/journal.pone.0235597)
Supplement: S1 Table — (DOCX) [file pone.0235597.s003.docx]

**S1 Table. Consequences of the Agrimonde-Terra scenarios on land use, food security and nutrition**

|  | **Metropolization** | | **Regionalization** | | **Households** | **Healthy** | | **Communities** | |
| --- | --- | --- | --- | --- | --- | --- | --- | --- | --- |
| 2010- 2050 world change (10^6^ ha)  **Agricultural land**  **Cropland**  **Pastureland** | Ultrap  -54  [-1%]  +243 [+16%]  -297  [-9%] | Animp  +1 318 [+27%]  +620 [+40%]  +698 [+21%] | techno A  +249 [+5%]  +70 [+4.5%]  +179 [+5.5%] | techno B  +691 [+14%]  +174 [+11%]  +517 [+15%] | Non available | techno C  +29 [+0.6%]  -56  [-4%]  +85 [+2.5%] | techno D  +269 [+5.5%]  +50  [+3%]  +219 [+6.5%] | AE  +142  [+3%]  +277 [+18%]  -135  [-4%] | Collapse  +2 013 [+41%]  +555 [+36%]  +1 458 [+43.5%] |
| ***Availability*** | Strong increase for developing c., but under tension due to large expansion of agricultural land (Animp variant) in all regions except Rest of America, FSU*, China | | Global improvement in terms of caloric availability, but tensions in some regions because of scarcity of land (India, WA*, UE for arable land; WA and Rest of Africa* for pasture). | | Stable [estimation] | Improvement for developing countries, but potential conflicts between food availability and mitigation of climate change in some regions (tensions on arable land in India and WA; on pasture in Rest of Africa). | | Decrease due to land shortages, low yields and livestock performances in all regions, tension due to strong increase of arable land and pasture in all regions except UE and China. | |
| ***Access*** | International food markets  Increase of average income with global development, but significant inequalities  Better physical access with supermarkets, but ‘food deserts’ | | Regional food markets  Increase of rural incomes linked to rural and agrifood development  Better physical access through rural markets | | Local and international markets, informal food exchanges  Increase of rural incomes due to non-farm employment development  Better physical access through mobility infrastructures | Different types of markets  Access to quality and fresh products subsidized  Access to diversified foods for rural households with crop diversification  Reinforcement of open air markets in developing countries | | Informal markets and food solidarities  Decreasing incomes in urban areas. Low physical access due to weak food markets  Better access in rural areas with commons, but low access if population increase or degraded land | |
| ***Utilization*** | Food ultra-processing  Increase of ready-to-eat products, street foods and sodas. Decrease of cooking practices | | Regional food processing in medium-size cities  Cooking practices with regional products. Culinary culture | | Industrial and small-scale food processing  Hybrid style of eating: traditional cooking, and ready-to-eat or street foods for mobile people | Improved quality of industrial food processing (fiber and nutrients)  Education to health and cooking | | Local and small-scale food processing  Traditional cooking  Street food in urban areas | |
| ***Stability*** | Instability of international markets due to pressure on land and climate change | | Regional instability when unstable climate conditions | | Occasional instability if conflicts arise within food networks | Stability through market rules but depending on public incentives | | Strong instability due to local climatic conditions and weakness of food markets | |
| ***Nutrition*** | Strong impacts of unhealthy foods and overconsumption: increase of NCD and overnutrition  Double burden of malnutrition in some developing countries. | | Reduced undernutrion (increase of animal products in developing countries)  Limited impact on health of ultra-processed foods | | Undernutrion decrease through higher food diversity  But impact on overnutrition of increased consumption of ultraprocessed foods | Decrease of malnutrition and NCD through strong food diversification and moderate intakes in animal products | | Decrease overnutrition in developed countries because of the reduced caloric consumption  Undernutrition decrease with diet diversity through agroecology  Undernutrition increase with survival agriculture | |

* FSU (Former Soviet Union), WA (Western Africa), Rest of Africa (ECS Africa + North Africa)
