# Supplementary material for: Exploring the future of land use and food security: A new set of global scenarios
Source: PLoS One. 2020 Jul 8;15(7):e0235597. doi: 10.1371/journal.pone.0235597 (PMC7343151; doi:10.1371/journal.pone.0235597)
Supplement: S1 Fig — (DOCX) [file pone.0235597.s004.docx]

**S1 Fig. Regional agricultural land area in the initial 2010 situation and in 2050 in the various scenarios (Million hectares)**

**S1 Fig a. Cropland**

**
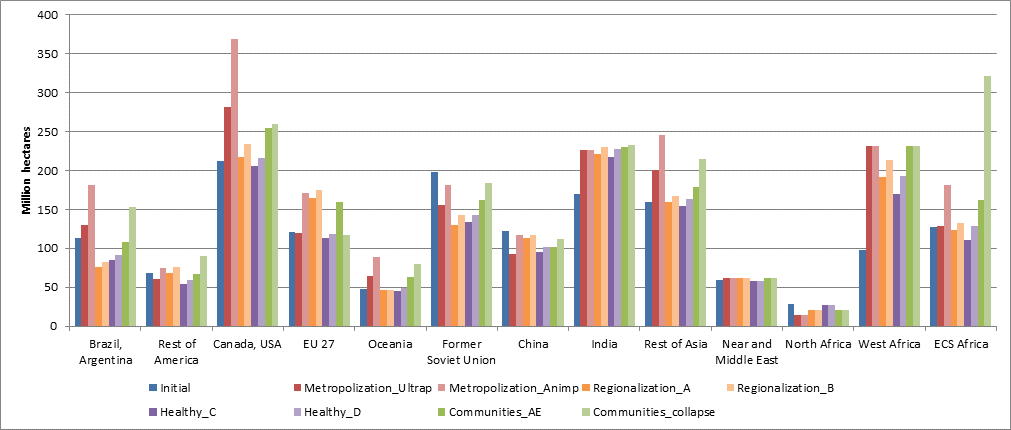
**

**S1 Fig b. Pastureland**

**
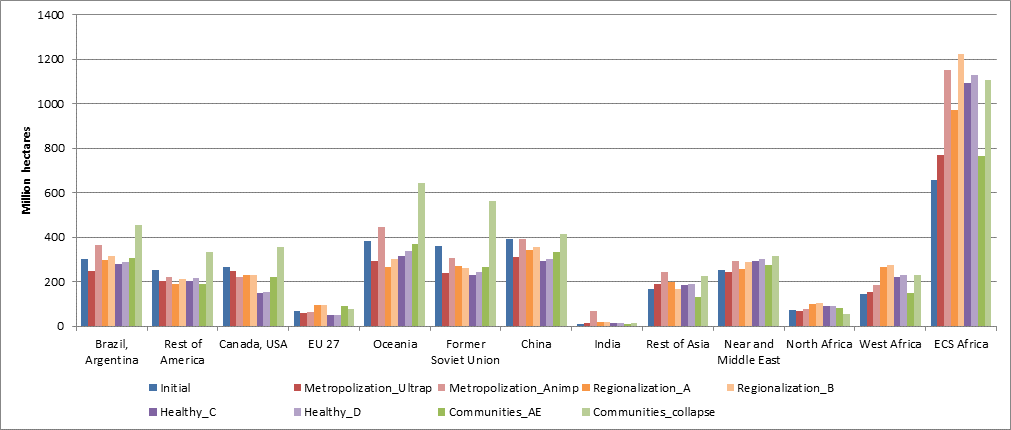
**

*Source: GlobAgri-AgT simulation results*
